# Molecular Mechanisms of Epileptic Encephalopathy Caused by KCNMA1 Loss-of-Function Mutations

**DOI:** 10.3389/fphar.2021.775328

**Published:** 2022-01-13

**Authors:** Yu Yao, Dongxiao Qu, Xiaoping Jing, Yuxiang Jia, Qi Zhong, Limin Zhuo, Xingxing Chen, Guoyi Li, Lele Tang, Yudan Zhu, Xuemei Zhang, Yonghua Ji, Zhiping Li, Jie Tao

**Affiliations:** ^1^ School of Medicine and School of Life Sciences, Shanghai University, Shanghai, China; ^2^ Department of Neurology and Central Laboratory, Putuo Hospital, Shanghai University of Traditional Chinese Medicine, Shanghai, China; ^3^ Department of Traditional Chinese Medicine, Shanghai Children’s Hospital, Shanghai Jiao Tong University, Shanghai, China; ^4^ Department of Pharmacology, School of Pharmacy, Fudan University, Shanghai, China; ^5^ Department of Clinical Pharmacy, Children’s Hospital of Fudan University, National Children’s Medical Center, Shanghai, China

**Keywords:** BK channel, KCNMA1, loss-of-function variants, epilepsy, neuroinflammation, autophagy

## Abstract

The gene *kcnma1* encodes the α-subunit of high-conductance calcium- and voltage-dependent K^+^ (BK) potassium channel. With the development of generation gene sequencing technology, many KCNMA1 mutants have been identified and are more closely related to generalized epilepsy and paroxysmal dyskinesia. Here, we performed a genetic screen of 26 patients with febrile seizures and identified a novel mutation of KCNMA1 (E155Q). Electrophysiological characterization of different KCNMA1 mutants in HEK 293T cells, the previously-reported R458T and E884K variants (not yet determined), as well as the newly-found E155Q variant, revealed that the current density amplitude of all the above variants was significantly smaller than that of the wild-type (WT) channel. All the above variants caused a positive shift of the I-V curve and played a role through the loss-of-function (LOF) mechanism. Moreover, the β4 subunit slowed down the activation of the E155Q mutant. Then, we used *kcnma1* knockout (BK KO) mice as the overall animal model of LOF mutants. It was found that BK KO mice had spontaneous epilepsy, motor impairment, autophagic dysfunction, abnormal electroencephalogram (EEG) signals, as well as possible anxiety and cognitive impairment. In addition, we performed transcriptomic analysis on the hippocampus and cortex of BK KO and WT mice. We identified many differentially expressed genes (DEGs). Eight dysregulated genes [i.e., (Gfap and Grm3 associated with astrocyte activation) (Alpl and Nlrp10 associated with neuroinflammation) (Efna5 and Reln associated with epilepsy) (Cdkn1a and Nr4a1 associated with autophagy)] were validated by RT-PCR, which showed a high concordance with transcriptomic analysis. Calcium imaging results suggested that BK might regulate the autophagy pathway from TRPML1. In conclusion, our study indicated that newly-found point E155Q resulted in a novel loss-of-function variant and the dysregulation of gene expression, especially astrocyte activation, neuroinflammation and autophagy, might be the molecular mechanism of BK-LOF meditated epilepsy.

## Introduction

Ion channels are expressed throughout the body and perform important physiological functions, such as neuronal excitability and the tone of smooth muscle. Ion channel disease, also known as ion channelopathy, is unusually considered to be caused by the gene mutation and abnormal function of ion channel subunits (Zheng and Trudeau, 2015; Bailey et al., 2019). BK channel is widely expressed in neurons and muscles (Fagerberg et al., 2014), and is also related to functions such as membrane potential repolarization, neuronal excitability control, neurotransmitter release, innate immunity, and cochlear hair cell regulation (Petersen and Maruyama, 1984; Murrow and Fuchs, 1990; Brayden and Nelson, 1992; Robitaille and Charlton, 1992).

Human *kcnma1* encodes the α-subunit of high-conductance calcium- and voltage-dependent K^+^ (BK) potassium channel. The α subunit of BK channel contains seven transmembrane fragments (S0-S6) and a large intracellular COOH terminal, consisting of two RCK domains (responsible for calcium sensing through the high-affinity Ca^2+^ binding sites), and S1-S4 acts as the voltage sensor, S5 and S6 fragments as well as P-loop form the pore region of the channel, and the (TVGYG) sequence of S6 is considered as a selective filter for potassium ions (Latorre et al., 2017). BK channel is allosterically activated by the changes of not only intracellular calcium concentration, but also membrane potential. The main sources of BK channel dysfunction are *de novo* and genetic nucleotide changes, which are roughly divided into gain-of-function (GOF) and loss-of-function (LOF) (Bailey et al., 2019). LOF mutation changes the channel activity by reducing the current amplitude or duration, while GOF mutation activates faster, increases Ca^2+^ sensitivity and current amplitude (Du et al., 2005; Moldenhauer et al., 2020).

In 2005, the abnormality of the BK channel was associated with human diseases for the first time. The substitution of aspartic acid at position 434 of the alpha subunit of BK channel by glycine would induce repolarization of action potentials, accelerate the firing rate, and increase the overall excitability of neurons, leading to systemic epilepsy (Du et al., 2005). Interestingly, the GOF phenotype of D434G mutant is due to increased BK channel Ca^2+^ sensitivity (Du et al., 2005), but in N995S (also called N999S and N1053S) mutants, the mechanism for BK GOF is the left shift of conductance-voltage (G-V) curve (Moldenhauer et al., 2020). However, different from the phenotype of the mutants above, BK channel C413Y, P805L, D984N (Bailey et al., 2019) and G354S-LOF (Du et al., 2020) mutations have been found to reduce the channel currents, with varying presence of seizures, dyskinesia, and dystonia (Bailey et al., 2019). In addition to the genetic mutation and abnormal expression of the channel itself, the physiological characteristics of the BK channel are also affected by the interaction of its α subunit and auxiliary subunits (e.g., β1-4 subunits or γ1-4 subunits). The β4 subunit is an auxiliary subunit specifically expressed by neurons, dominantly expressed in brain. The BK channel composed of it and the α subunit activates relatively slowly than the channel composed of only the α subunit. The mice lacking the β4 subunit show significant symptoms of temporal lobe epilepsy (Brenner et al., 2005). In addition, mutations in the β3 subunit also cause epilepsy (Lorenz et al., 2007).

The unique physiological behavior of the BK channel enables it to both enhance and reduce the excitability of neurons. Therefore, the role of BK channel in the pathogenesis of epilepsy is still controversial and is an increasingly intense research field (Zhu et al., 2018).

Here, we report a novel *de novo* KCNMA1 mutant (E155Q) in a patient. Electrophysiological results show that E155Q, R458T (not yet determined), and E884K (not yet determined) mutant all present a LOF phenotype. A series of behavioral experiments show that BK KO mice (as the overall animal model of LOF) have spontaneous epilepsy, motor impairment, abnormal electroencephalogram (EEG) signals, autophagic dysfunction, as well as possible anxiety and cognitive impairment. This study expands the mutation spectrum of KCNMA1-epilepsy, explores the possible mechanism of epilepsy by transcriptome, reveals the relationship between KCNMA1-LOF and epilepsy, and provides a possible molecular template for individualized treatment of epilepsy.

## Methods

### Mutation Screening

Genomic DNA was extracted from peripheral blood with kit (DP348, Tiangen, Beijing, China). The samples were assessed by Shanghai Biotechnology Corp, China. KCNMA1 variants were examined in children with febrile seizures by whole-exome sequencing. This study was approved by the Institutional Review Board at Children’s Hospital of Fudan University, Shanghai (National Children’s Medical Center, Fudan University). And informed consent was given by the parent or guardian.

### Site-Directed Mutagenesis of KCNMA1 (hBKα) Plasmids

The plasmids containing hSloα (U23767) and β4 (KCNMB4; AF 160967.1) were gifts from J.D. Lippiat (Leeds university) (Lippiat et al., 2003). Three individual point-mutations (E155Q, R458T, and E884K) were constructed. Each amino-acid substitution was introduced into the hSloα plasmid using a Hieff Mut™ Site-Directed Mutagenesis Kit (11004ES10, Yeasen, Shanghai, China) according to the manufacturer’s protocol. Site-directed mutagenesis was performed with the following primers [P1: 5′-CCA​GCC​GAC​CTG​GGC​GGC​CAC​TG-3′, P2: 5′-CAG​TGG​CCG​CCC​AGG​TCG​GCT​GG-3′ (E155Q); P1: 5′-CCA​CCT​GAG​TAA​AAT​GTG​TTT​TGA​ACA​GAG​CTT​CAA​GCT​CCA​G-3′, P2: 5′-CTG​GAG​CTT​GAA​GCT​CTG​TTC​AAA​ACA​CAT​TTT​ACT​CAG​GTG​G-3′ (R458T); P1: 5′-GAC​GCC​AAG​ATG​CAT​TTC​TTG​TCC​TGC​AGC​GAA-3′, P2: 5′-TTC​GCT​GCA​GGA​CAA​GAA​ATG​CAT​CTT​GGC​GTC-3′ (E884K)]. All mutant constructs were verified by sequencing (GENEWIZ, Jiangsu, China).

### Cell Culture and Transfection

All experiments were performed on HEK 293T cell lines. HEK 293T cells were obtained from Shanghai cell bank of Chinese Academy of Science. The cells were both cultured in Dulbecco’s modified Eagle medium (DMEM; Life Technologies, Grand Island, NY) supplemented with 10% heat-inactivated fetal bovine serum (FBS; Gibco, Grand Island, NY). Culture dishes were incubated at 37°C in a humidified atmosphere containing 5% CO_2_, and subcultured approximately every 2–3 days. One day before transfection, HEK 293T cells were transferred to 24 well plates. At 90% confluence, cells were transiently transfected using Lipofectamine-3000 (Invitrogen, United States) at a ratio of 2 µl reagent with 1 µg total plasmid per well. Electrophysiological recordings from fluorescent cells were made 48 h after transfection.

### Electrophysiological Recordings

Whole-cell voltage-clamp experiments were performed following the procedures described previously (Hamill et al., 1981), using an EPC-9 amplifier (HEKA Eletronik, Germany) at room temperature (21–25°C). Patch pipettes were fabricated from glass capillary tubes by PC-10 Puller (Narishige, Japan) with the resistance of 2–3 MΩ. Data acquisition and stimulation protocols were controlled by a Pentium III computer (Legend, Beijing, China) equipped with Pulse/PulseFit 8.3 software (HEKA Eletronik, Germany). Capacitance transients were cancelled. Cells with a seal resistance (Rseal) below 1 GΩ were omitted. Series resistance (Rs) was compensated (80–85%) to minimize voltage errors, and cells with an uncompensated Rs above 10 MΩ were omitted. Leak subtraction was performed using P/6 protocol. Data were low-passed at 10 kHz. Unless stated specially, for HEK 293T cells, the holding potential was −80 mV. BK channel currents were elicited by the step pulses ranging from −100 to +150 mV for 200 ms with the increments of 10 mV. The holding potentials were held at −80 mV for BK channel. Current density calculation formula (pA/pF), where pA represents the current of BK channel and pF represents the membrane area of measured cell.

### Solutions

In the patch-clamp recordings, the standard bath solution for HEK 293T cells was consisted of the following components (in mM): NaCl 135, KCl 5, MgCl2·6H2O 1, CaCl2 1.8, HEPES 10, glucose 10 (pH 7.4 with NaOH). Pipette solutions for HEK 293T cells were composed of the following components (in mM): NaCl 10, KCl 117, MgSO4 4, HEPES 10, EGTA 1 (pH 7.2 with KOH). The total Ca^2+^ to be added to give the desired free concentration was calculated using the program WEBMAXC STANDARD (https://somapp.ucdmc.ucdavis.edu/pharmacology/bers/maxchelator/webmaxc/webmaxcS.htm).

### Animals and Genotyping

The BK knockout (BK KO, *kcnma1* ^−/−^) mice were established by breeding BK^+/−^ males and females. These breeding pairs provided wild-type (*kcnma1* ^+/+^), heterozygous (*kcnma1* ^+/−^) and BK KO (*kcnma1* ^−/−^) littermates (Wang et al., 2019), and three-month-old (10–12 weeks) male BK KO mice were subjected to subsequent experiments, such as gait analysis, morris water maze, field potential recordings, etc. The *kcnma1* knockout was generated by a frameshift mutation (− 16bp) in exon 4. Genotyping was performed with the following primers: P1: 5′-CTT​CCT​GCT​TGT​CCT​TCC​TC-3′, P2: 5′-CAT​TGC​TTC​AAA​CCC​TTC​CT-3′, and the PCR products were directly sequenced. In this study, all animals were randomly fed and watered in SPF standard animal facilities and were fed and watered in 21°C, 50% humidity, 12 h light: under the schedule of 12 h dark, five animals were raised in each cage, and all the animal work was carried out under the moral permission of the ethics review group of Shanghai University of traditional Chinese Medicine.

### FP Recordings

Four BK KO and WT male mice were implanted with electrodes for the field potential (FP) recordings. At the age of 3 months (10–12 weeks), the animals were first anaesthetized by pentobarbital sodium [in a dose of 40 mg/kg through intraperitoneal injection (i.p.)]. Then, they were put in a stereotaxic frame (Narishige, Tokyo, Japan). Their heads were shaved and sterilized with povidone-iodine. In order to expose the sagittal, coronal, and lambdoid sutures, cut the scalp along the center. Subsequently, the recording electrode was also inserted into the lateral dorsal hippocampus (AP −2.0 mm posterior to bregma, V 1.5 mm ventral to the dura surface, and L 1.5 mm lateral to the skull midline). The reference wire is located in the electrode bundle, and the grounding electrode is placed at the front of the skull. The wound surface was sealed with dental cement. The electrode was fixed simultaneously. Data were collected only for animals with accurately positioned electrode. The FP recorded after the mice were awake. The FP signals together with synchronized video could be recorded with the OmniPlex (Plexon, United States). The mount of the head was linked to a preamplifier which is tied with the analog-digital converter box. According to Nesquet’s sampling theory, take 1 Hz as the sampling frequency of local FP recording and set 50 Hz high-pass filter and 300 Hz low-pass filter to record for more than 30 min continuously. The results of local FP recording were exported as the *.pl2 file format, and offline sorter v4 software was used for visualization preview. Local FP analysis selected the same channel through MATLAB (MathWorks, United States) program to export data. The wavelet transform is used to decompose the signal of different frequencies of local FP and get the physiological rhythm of different frequencies (δ: from 0 to 4 Hz, θ: from 4 to 8 Hz, α: from 8 to 13 Hz: β: from 13 to 30 Hz, and γ: from 30 to 100 Hz). The Welch method, hamming window, and fast Fourier transform method were used to calculate the frequency domain information of the local FP in power spectrum analysis. The time domain of energy change is calculated by weighted operation. The PSD calculations follow the formula given below.

$$\overset{+\infty }{\underset{-\infty }{\int }}x^{2}\left(t\right)\mathrm{dt}=\frac{1}{2\pi }\overset{+\infty }{\underset{-\infty }{\int }}{\left|X\left(\mathrm{jw}\right)\right|}^{2}d\omega$$

$$\text{P}=\underset{\text{T}\to \infty }{\lim}\frac{1}{2T}\overset{T}{\underset{-T}{\int }}x^{2}\left(t\right)\mathrm{dt}=\frac{1}{2\pi }\overset{+\infty }{\underset{-\infty }{\int }}\underset{T\to \infty }{\lim}\frac{1}{2T}{\left|X_{T}\left(\omega \right)\right|}^{2}d\omega$$

### Behavioral Observation of Epilepsy

In order to eliminate the possibility of the human error, behavioral observations are double-blind during experiments. The Racine’s five-point scale (Racine, 1972), improved by Fathollahi et al. (1997), was employed to classify the seizure-like behavior at different stages. Stage 0 is termed no response; stage 1 is called facial and ear twitching; stage 2 is myoclonic jerks without an upright position; stage 3 represents myoclonic jerks and upright position with bilateral forelimb clonus; stage 4 stands for clonic-tonic seizure; stage 5 is named generalized clonic-tonic seizures and loss of postural control. The severity of seizures in BK KO mice was evaluated according to the grade and time of seizures. The least interval between two countable seizures was set as 5 s during the quantification of all seizure numbers.

### Immunofluorescence Staining

Frozen sections were permeabilized with 0.5% Triton X-100, and blocked for 1 h at room temperature (RT) with 5% bovine serum albumin. Without washing, sections were incubated overnight at 4°C with primary antibodies. These include against LC3B (1:200 dilution; ab48394; Abcam), LAMP1(1:500 dilution; ab25630; Abcam), and Iba-1 (1:500 dilution; ab178846; Abcam). washing four times in PBS (4 × 5 min), sections were incubated with goat anti-rabbit antibody conjugated with Alexa Fluor 594 (for LC3B; 1:200 dilution; ab150080; Abcam); goat polyclonal secondary antibody to mouse conjugated with Alexa Fluor 488 (for LAMP1; 1:200 dilution; ab150113; Abcam) and Alexa Fluor 350-labeled goat anti-rabbit IgG (H + L) (for Iba-1; 1:500 dilution; A0408; Beyotime) for 1 h at RT, washing four times in PBS (4 × 5 min). Slices were cover-slipped with 50% glycerin. Fluorescence images were captured using a Virtual/Digital Slice Microscope (Olympus, Tokyo, Japan). Quantification was performed using ImageJ software.

### Calcium Imaging

HEK293T cells, co-transfected with pcDNA3-TRPML1-GCaMP3, gifted from Prof. Xu HX (University of Michigan, United States), were kept in HBSS (Hank’s balanced salt solution) at RT for 30 min. Confocal imaging was performed by using a Zeiss confocal LSM 880, a laser scanning microscope system (Zeiss, Germany). GCaMP3 was evoked with a laser wavelength at 488 nm, and the fluorescence images were collected with the resolution of 512 × 512 pixels. The ROI (regions of interest; 3 × 3 pixels) were selected in individual HEK293T cells by Zeiss LSM Image Browser (Zeiss, Germany) to track the changes in the ratio of fluorescence intensity. The ratio (F/F0) of fluorescence intensity was calculated by dividing fluorescence intensity at time t (F) with the beginning fluorescence intensity (F0) of the experiment.

### Y-Maze Test

Y‐maze test was performed as previously described (Watanabe et al., 2011). Exploratory activity was measured using a Y‐maze apparatus (arm length: 40 cm, arm bottom width: 10 cm, arm upper width: 10 cm, height of wall: 12 cm). The floor and the wall of the maze is made of black PVC plastic. Each subject was placed in the arm Ⅰ of the Y‐maze field. The alteration (%) and total distance (m) was recorded using a modified version of the image software. Data were collected for a period of 10 min.

### Gait Analysis

We analyzed gait of the mice during walk/trot locomotion by ventral plane videography as described (Hampton et al., 2004; Koshimizu et al., 2014), using digigait imaging system (Mouse Specifics Inc.). This system enables mice to walk on a motorized transparent treadmill belt, and the software automatically identifies the stance and swing components of stride and calculates stride length, print area, mean intensity, and swing speed. Briefly, we placed the mice on a treadmill belt that moves at constant speed. We collected digital video images of mice.

### Morris Water Maze Test

A dark blue pool (diameter: 120 cm, depth: 50 cm) was placed in a quiet room and filled with white-colored water. The water was equilibrated to room temperature (between 22 and 23°C) before the MWM test. Colored papers with a variety of different shapes were posted around the pool as visual cues. A platform of 10 cm in diameter was used. For hidden platform trials, the platform was positioned 1.5 cm below water surface. The MWM test was performed in the period of 11AM—3PM to minimize circadian effects. The BK KO and control mice were tested in same days, and testing sequences for individual mice were altered in each test day. A protocol with 5 days of visible platform trials and 1 day of hidden platform trials were employed. In the trials, individual mice underwent four trials per day, and the maximal time for each trial was 60 s. If mice did not find the platform within 60 s, they were guided to the platform by the experimenter’s hand and allowed to stay on the platform for 60 s. For the probe tests in which the platform was removed from the pool, individual mice underwent a trial of 60 s in each quadrant. If mice exhibited convulsions shortly before or during a trial, they were allowed to recover for 20–30 min before next trial. Any trial interfered with convulsions were excluded from analysis. Frequency and the time in aim-quadrant during the probe trials were analyzed. Group data for the BK KO and WT mice were compared.

### Open Filed Test

The open field behavior experiment device is a very effective device for measuring spontaneous and exploratory behaviors to measure the degree of anxiety in mice. Place the animal in an unknown environment with walls around it, and the rodents will spontaneously tend to move on the edges rather than the open center of the area. In this experiment, the device is composed of a black polystyrene box (50 × 50 × 50 cm), which is divided into two areas: the outer square (periphery) and the inner square (center). Each mouse was placed in the center of the box and explored freely for 10 min. Observe their behavior through the animal video monitoring with the behavior software, and measure the stay time (s), the total distance of movement in the central area (m), the average speed (mm/s) through the computer tracking system, evaluate spontaneous and exploratory behavior.

### Tissue Sample Collections

Three different BK KO and WT adult mice were randomly sampled, sacrificed, and their cortices and hippocampi extracted under 2-min, frozen, and stored at − 80°C.

### RNA Extraction

Total RNA was extracted from the mouse cortex/hippocampus using TRIzol Reagent (15596018, Invitrogen) according to the manufacturer’s protocol. Using the a nanodrop (Thermo Scientific NanoDrop 2000c Spectrophotometer), the RNA concentration of each sample was determined by measuring the absorbance at 260 nm (A260), and its purity was determined by the ratio of the absorbance measured at 260 nm (A260) and 280 nm (A280). The ratios of A260/A280 between 1.9 and 2.1 were considered acceptable.

### Library Preparation for Transcriptome Sequencing

A total amount of 2 μg RNA per sample was used as input material for the RNA sample preparations. Sequence libraries were generated using NEBNext UltraTM RNA Library Prep Kit for Illumina (NEB, United States) following manufacturer’s recommendations and index codes were added to attribute sequences in each sample. Briefly, mRNA was purified from total RNA using poly-T oligo-attached magnetic beads. Fragmentation was carried out using divalent cations under elevated temperature in NEBNext First Strand Synthesis Reaction Buffer (5×). First strand cDNA was synthesized using random hexamer primer and M-MuLV Reverse transcriptase (RNase H). Second strand cDNA synthesis was subsequently performed using DNA Polymerase I and RNase H. Remaining overhangs was converted into blunt ends to exonuclease/polymerase activities. After adenylate of 3′ ends of DNA fragments, NEBNext Adaptor with hairpin loop structure was ligated to prepare for hybridization. In order to select cDNA fragments of preferentially 200–250 bp in length, the library fragments were purified with AMPure XP system (Beckman Coulter, Beverly, United States). Then 3 μl USER Enzyme (NEB, United States) was used with size-selected, adaptor-ligated cDNA at 37°C for 15 min followed by 5 min at 95°C before PCR. Then PCR was performed with Phusion High-Fidelity DNA polymerase, Universal PCR primers and Index (X) Primer. At last, PCR products were purified (AMPure XP system) and library quality was assessed in the Agilent Bioanalyzer 2,100 system.

### Clustering and Sequencing

The clustering of the index-coded samples was performed on a cBot Cluster Generation System using TruSeq PE Cluster Kit v4-cBot-HS (Illumia) according to the manufacturer’s instructions. After cluster generation, the library preparations were sequenced on an Illumina Hiseq 4,000 platform and paired-end 150 bp reads were generated.

### Differential Expression Genes Analysis

Differential expression analysis of two conditions/groups was performed using the DESeq R package (1.10.1). DESeq2 provides statistical routines for determining differential expression of digital gene expression data using a model based on the negative binomial distribution. The resulting *p* values were adjusted using the Benjamini and Hochberg’s approach to controlling the false discovery rate. Genes with pvalue <0.05 and |log2FC| > 0.58 found by DESeq were assigned as differentially expressed.

### GO Enrichment Analysis

Gene Ontology (GO) enrichment analysis of the DEGs was implemented by the GOseq R packages based Wallenius non-central hyper-geometric distribution, which can adjust for gene length bias in DEGs.

### KEGG Pathway Enrichment Analysis

KEGG is a database resource for understanding high-level functions and utilities of the biological system, such as the cell, the organism and the ecosystem, from molecular level information, especially large-scale molecular datasets generated by genome sequence and other high-throughput experimental technologies (http//:www.genome.Jp/kegg/) (Jiang et al., 2018). We used KOBAS (Mao et al., 2005) software to test the statistical enrichment of differential expression genes in KEGG pathways.

### PCR Primer Design and Testing

We first looked through NCBI’s Primer-Bank (https://pga.mgh.harvard.edu/primerbank/) to find PCR primers for selected genes. Primers were checked for specificity using NCBI’s Primer-BLAST program (https://www.ncbi.nlm.nih.gov/tools/primer-blast/) against RefSeq RNA to ensure no non-specific matches. When designing primers that could match multiple transcript variants, their sequences were aligned in ClustalW (http://www.genome.jp/tools/clustalw/), and only the primers that amplified a consensus region were used. All PCR primers were purchased from GENEWIZ (Jiangsu, China) and listed in Supplementary Table S3 with sizes of the resulting PCR products in base pairs (bp).

### Real-Time RT-PCR Validation of Selected DEGs

To validate the transcriptome gene expression data, six DEGs identified by transcriptome were validated by real-time RT-PCR using QuantityNova SYBR Green PCR Kit (208052, Qiagen, Valencia, CA). RT- PCR was performed in a one-step RT-PCR process according to the protocol on Roche480II using 30 ng RNA. Housekeeping gene β-actin was used as endogenous control. RNA was first reverse transcribed into cDNA at 65°C for 5 min. After enzyme activation at 95°C for 2 min, PCR was carried out at 95°C for 5 s and 60°C for 10 s for 45 cycles. For RT-PCR analysis, each sample was run in triplicates. Comparative Ct method (delta Ct method) was used to calculate the fold differences between BK KO and WT groups.

### Statistical Analysis

Data were analyzed with Origin 8.5 (OriginLab, United States), Excel 2016 (Microsoft, WA) and Prism 6 (GraphPad software, San Diego, CA). Data are presented as the mean ± standard error of the mean (SEM). Student’s t-test or one-way ANOVA was used to assess the statistical significance of differences. When *p* < 0.05, differences were accepted as significant.

## Results

### Identification of a *de novo* Variant in KCNMA1 (hbkα) From a Patient With Febrile Seizures

In this study, among the 26 clinical patients, two patients had a history of head trauma, two patients had a history of asphyxia and oxygen inhalation, two patients had premature delivery and one patient had severe pneumonia, which might be the inducement of epilepsy. In addition, eleven patients had abnormal EEG, five patients had abnormal MRI, and two patients had a family history of epilepsy (Table 1).

**TABLE 1:** Clinical data of 26 patients with one LOF variants in BK channels.

Patient ID genomic variant	2 p. E155Q	13 NA	4 p. E471G	20 c. 344A > G
Gene	KCNMA1	SCN1A	UPF3B	Panel ASXL3
*De novo* variant	Yes	NA	NA	NA
Gender	M	M	F	F
Age	6	6	17	3
Course (years)	5	5	8	2
Age of seizure onset (years)	1	1	9	1
Epilepsy details	Tonic-clonic limbs, unconsciousness, eyes rolled up and staring, no answer	Tonic-clonic limbs, unconsciousness, foaming at the mouth, lasting	Often onset in sleep and early morning, accompanied by disturbances in consciousness and urinary incontinence	Series of spasmodic seizures
		2–10min		
Anticonvulsant treatment	VPA	VPA, LEV, CLB, Ketogenic diet	OXC, VPA	VPA
EEG features	Sharp wave, sharp slow wave and spike slow wave. Rhythmic energy intensity of δ and θ increased	Sharp wave and sharp slow wave	A few sharp waves, sharp slow waves and spike slow waves	Peak rhythm disorder with intermittent phenomenon
MRI	NA	Normal	Normal	Brain dysplasia, left brain atrophy
Family history of epilepsy	No	No	No	Yes
Other details	Premature delivery 2/26			
	History of head trauma 2/26			
	History of severe pneumonia 1/26			
	History of suffocation and oxygen 2/26			
	Developmental delay 3/26			
	EEG abnormality 11/26			
	Abnormal MRI 5/26			
	Family history of epilepsy 2/26			

F, female; M, male; NA, data not available; VPA, valproate acid; LEV, levetiracetam; CLB, clobazam; OXC, oxcarbazepine.

**FIGURE 1:**
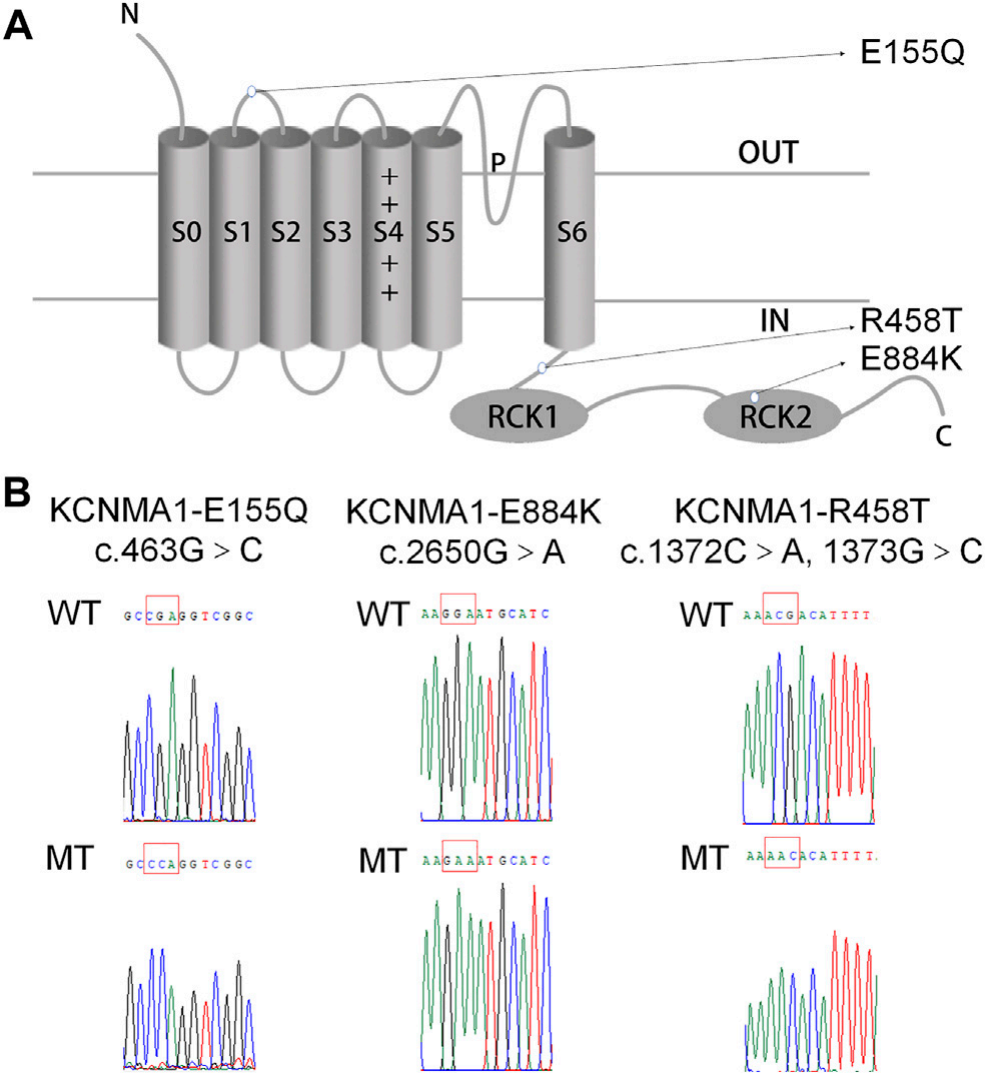
Location and sequencing of the KCNMA1 variants. **(A)** Predicted transmembrane topology of KCNMA1 depicting the location of the variants. **(B)** DNA sequencing identified the mutations in the constructed hBKα plasmid. The mutation sites are marked by a red square.

A novel variant c.463G > C [p.(E155Q)] in a patient with febrile seizures was identified. The patient, male, full-term born on August 26, 2015, bw3500 g, had a history of oligohydramnios and intrauterine distress, and no family history of epilepsy. He had several histories of febrile convulsions, which was characterized by tetanic clonus of limbs, unconsciousness, upturned eyes, unable to call, hyperactivity, repeated daze, poor language development and developmental delay. There was no abnormality in blood tandem mass spectrometry, and the energy of urine tandem mass spectrometry was disordered. EEG showed that there were some sharp waves, and spike waves on both sides of the brain. We predicted that the location of amino-acid E155 was in the domain S1/S2 linker of BK α (Figure 1A). A previous study already reported that R458T and E884K mutants were located in the domain S6/RCK1 cytoplasmic linker and the RCK2 domain of BKα (Figure 1A). Therefore, we constructed three plasmids: KCNMA1-E155Q, KCNMA1-R458T, and KCNMA1-E884K (Figure 1B).

### Electrophysiological Characteristics of BK Channel LOF Variant E155Q

**FIGURE 2:**
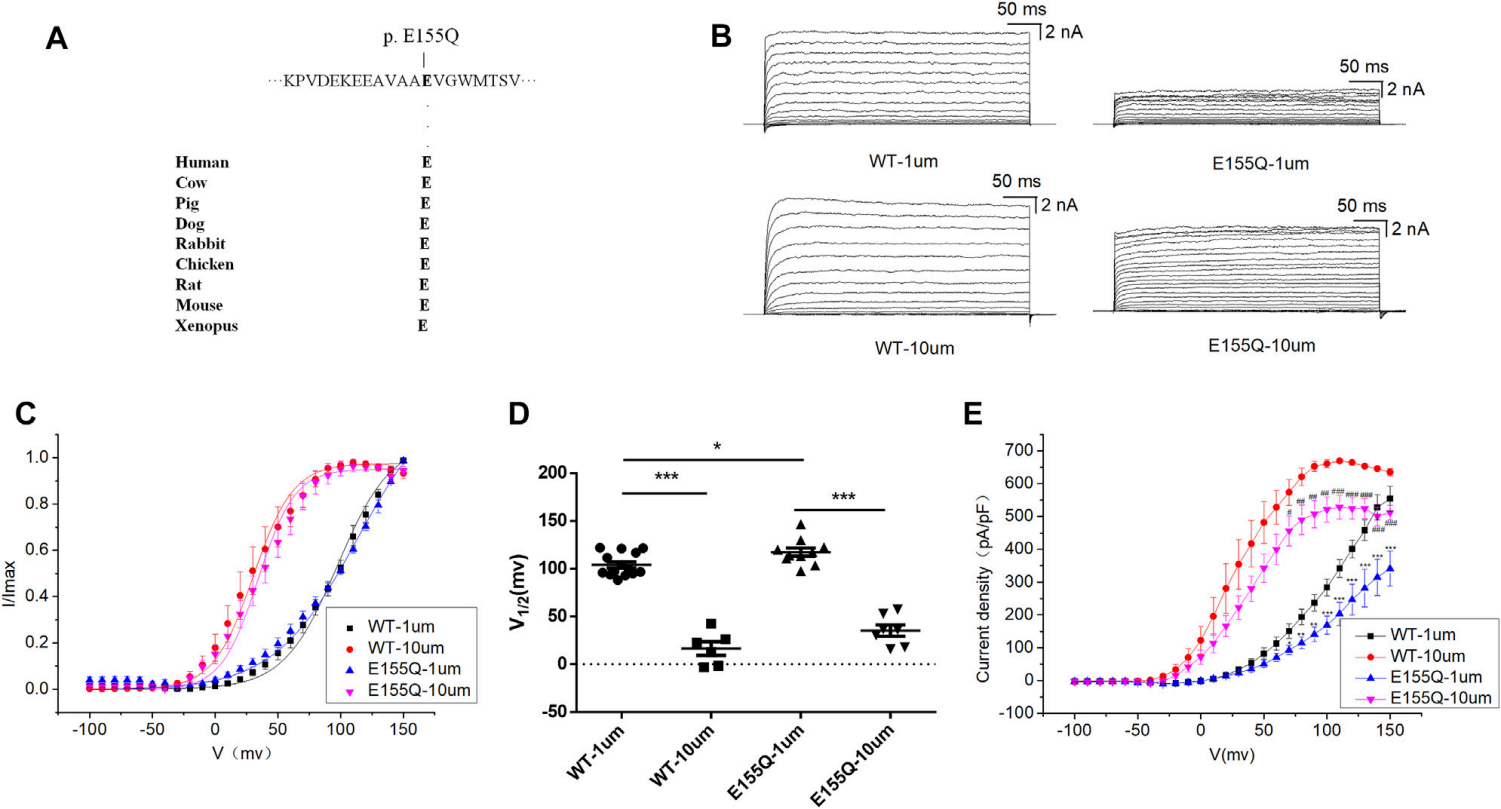
Electrophysiological characterization of variant E155Q. **(A)** The E155Q variant occur at an evolutionarily conserved amino acid residues. **(B)** Representative macroscopic currents of WT and mutant BK channels with variant E155Q from whole-cell patch experiments in the presence of 1 and 10 μm Ca^2+^. **(C)** The I-V curves of WT and E155Q mutant BK channels are shown at 1 and 10 μm Ca^2+^. The I-V curves are fitted by Boltzmann function (solid lines) with V1/2 and slope factor at nominal at 1 μm Ca^2+^ [97.5 ± 4.1 mV, 21.4 ± 2.1 WT, and 113.4 ± 6.4 mV, 33.2 ± 3.3 p.(E155Q)] and at 10 μm Ca^2+^ [30.4 ± 2.4 mV, 15.9 ± 0.8 WT, and 34.2 ± 2.9 mV, 14.6 ± 1.6 p.(E155Q)]. **(D)** Scatter plots of voltage at half-maximal activation (V1/2) for WT and variants. **(E)** The current density of WT and E155Q mutant BK channels are shown at 1 and 10 μm Ca^2+^. The data are presented as mean ± SEM. (Compared with WT-1um, *p < 0.05, **p < 0.01, ***p < 0.001. Compared with WT-10um, #*p* < 0.05, ##*p* < 0.01, ###*p* < 0.001. *n* = 6–14/group).

E155Q variant is highly conserved among different species during evolution (Figure 2A). To determine whether the c.463 G > C [p. (E155Q)] variant had an effect on BK channel function, we expressed mutant E155Q channel and control wild-type (WT) channel, recorded potassium currents, and analyzed the current voltage relationship. The results showed that in 1 and 10 μM free Ca^2+^ concentration, the macro currents amplitude of E155Q mutant was always smaller than that of WT (Figure 2B). Moreover, we found that the I-V curve of the mutant E155Q moved in the direction of positive potential (Figures 2C,D). Regardless of whether the free calcium concentration was 1 μM (*p* < 0.05 at 70 mv, *p* < 0.01 at 80–90 mv, *p* < 0.001 at 100–150 mv) or 10 μM (*p* < 0.05 at 70 mv, *p* < 0.01 at 80 mv −100 mv, *p* < 0.001 at 110 mv −150 mv), the E155Q mutant significantly reduced the current density of BK when the stimulation voltage reached 70–150 mv (Figure 2E, *n* = 6–14/group). Thus, the E155Q mutant is identified as a LOF variant.

### Effect of the β4 Subunit on the BK Channel Variant E155Q

The β4 subunit is an auxiliary subunit specifically expressed by neurons, dominantly expressed in brain. To determine whether the β4 subunit and the E155Q mutation interact, we co-transformed the β4 subunit and the E155Q mutant in HEK 293T cells. Compared with E155Q mutant, the β4 subunit had no effect on the macro current density amplitude, I-V curve, or V1/2 (Supplementary Figures S1A–D). Further analysis of the activation time constant (*τ*) revealed that *τ* of the E155Q+ β4 mutant was significantly greater than that of the E155Q mutant (Supplementary Figure S1E, *n* = 6–9/group), and thus the β4 subunit slowed down the activation of the E155Q mutant.

### Electrophysiological Characteristics of BK Channel LOF Variant R458T

**FIGURE 3:**
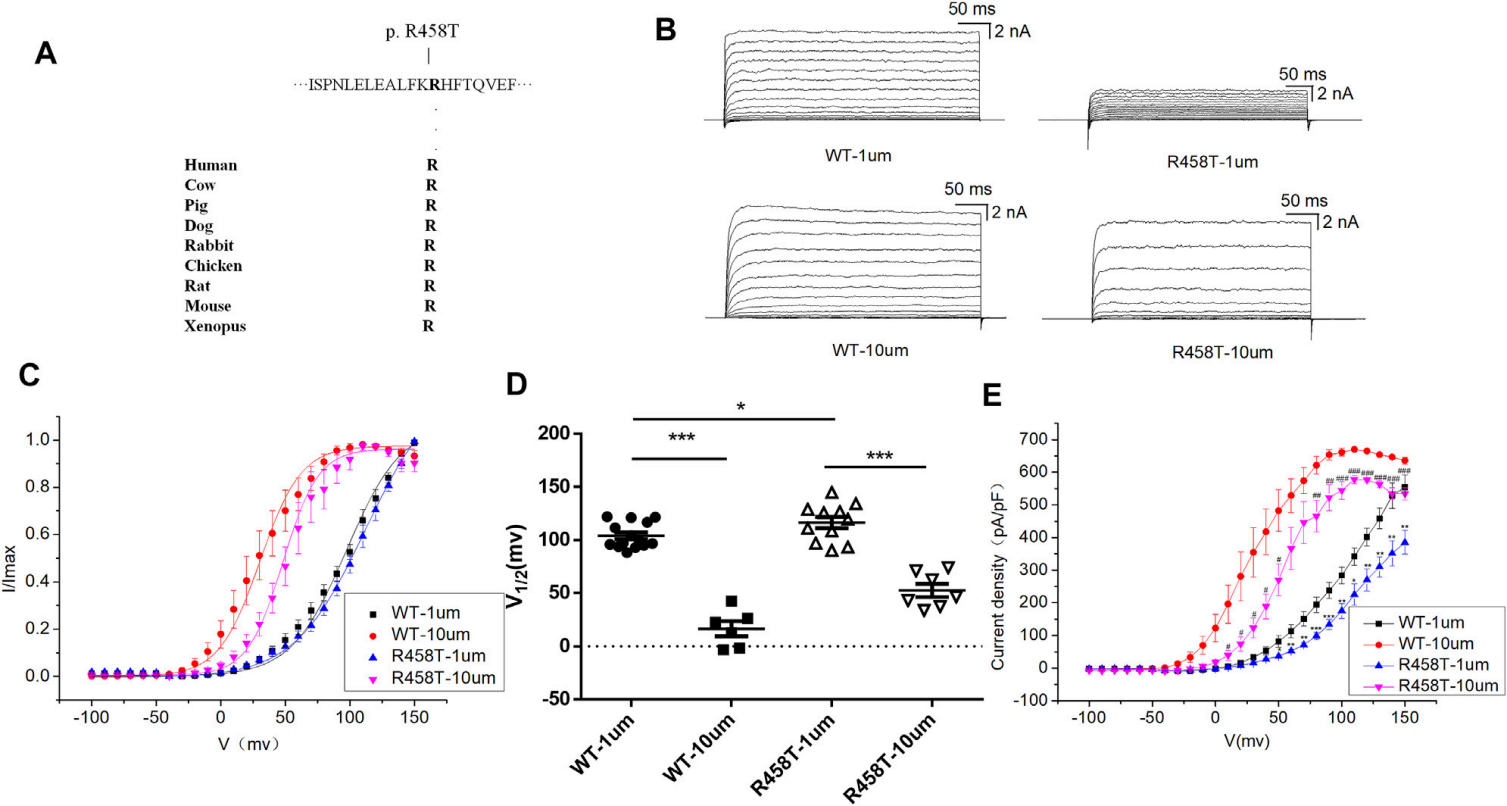
Electrophysiological characterization of variant R458T. **(A)** The R458T variant occur at an evolutionarily conserved amino acid residues. **(B)** Representative macroscopic currents of WT and mutant BK channels with variant R458T from whole-cell patch experiments in the presence of 1 and 10 μm Ca^2+^. **(C)** The I-V curves of WT and R458T mutant BK channels are shown at 1 and 10 μm Ca^2+^. The I-V curves are fitted by Boltzmann function (solid lines) with V1/2 and slope factor at nominal at 1 μm Ca^2+^ [97.5 ± 4.1 mV, 21.4 ± 2.1 WT, and 111.9 ± 6.3 mV, 27.5 ± 2.4 p.(R458T)] and at 10 μm Ca^2+^ [30.4 ± 2.4 mV, 15.9 ± 0.8 WT, and 48.2 ± 2.6 mV, 13.7 ± 1.1 p.(R458T)]. **(D)** Scatter plots of voltage at half-maximal activation (V1/2) for WT and variants. **(E)** The current density of WT and R458T mutant BK channels are shown at 1 and 10 μm Ca^2+^. The data are presented as mean ± SEM. (Compared with WT-1um, *p < 0.05, **p < 0.01, ***p < 0.001. Compared with WT-10um, #*p* < 0.05, ##*p* < 0.01, ###*p* < 0.001. *n* = 6–14/group).

R458T variant is highly conserved among different species during evolution (Figure 3A). We used the HEK 293T cell system to express and compare WT and mutant R458T channels. The results showed that R458T mutant significantly reduced the macro currents and current density amplitude of BK channel (Figures 3B,E). The I-V curve of R458T mutant shifted to the positive voltage direction (Figures 3C,D). Noticed, the smaller Ca^2+^-induced leftward shift of the I–V in the mutant suggests that its apparent Ca^2+^ sensitivity is less than that of the WT, further exacerbating the LOF phenotype.

### E884K Variant in the RCK2 Domain Markedly Reduced the Amplitude of the BK Currents

**FIGURE 4:**
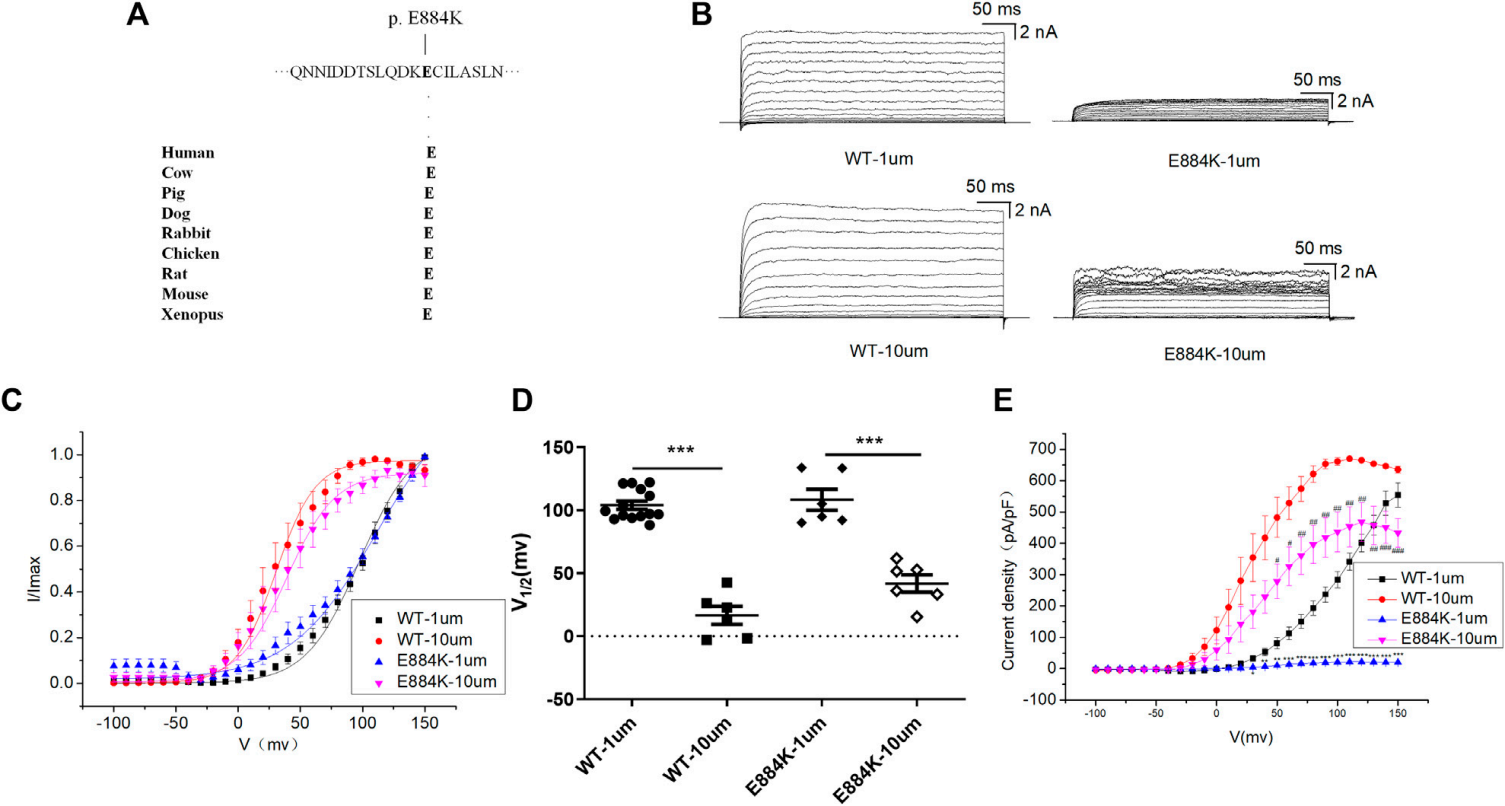
Electrophysiological characterization of variant E884K. **(A)** The E884K variant occur at an evolutionarily conserved amino acid residues. **(B)** Representative macroscopic currents of WT and mutant BK channels with variant E884K from whole-cell patch experiments in the presence of 1 and 10 μm Ca^2+^. **(C)** The I-V curves of WT and E884K mutant BK channels are shown at 1 and 10 μm Ca^2+^. The I-V curves are fitted by Boltzmann function (solid lines) with V1/2 and slope factor at nominal at 1 μm Ca^2+^ [97.5 ± 4.1 mV, 21.4 ± 2.1 WT, and 112.0 ± 6.7 mV, 34.8 ± 4.2 p.(E884K)] and at 10 μm Ca^2+^ [30.4 ± 2.4 mV, 15.9 ± 0.8 WT, and 39.3 ± 2.2 mV, 19.3 ± 1.4 p.(E884K)]. **(D)** Scatter plots of voltage at half-maximal activation (V1/2) for WT and variants. **(E)** The current density of WT and E884K mutant BK channels are shown at 1 and 10 μm Ca^2+^. The data are presented as mean ± SEM. (Compared with WT-1um, *p < 0.05, **p < 0.01, ***p < 0.001. Compared with WT-10um, #*p* < 0.05, ##*p* < 0.01, ###*p* < 0.001. *n* = 6–14/group).

E884K variant is highly conserved among different species during evolution (Figure 4A). The E884K variant significantly reduced the amplitude of the BK currents (Figure 4B). Moreover, the I-V curve of E884K variant shifted to the positive voltage direction similar to E155Q and R458T (Figures 4C,D). Regardless of whether the free calcium concentration was 1 μM (*p* < 0.01 at 50 mv, *p* < 0.001 at 60–150 mv) or 10 μM (*p* < 0.05 at 50–60 mv, *p* < 0.01 at 70–130 mv, *p* < 0.001 at 140–150 mv) the E884K mutant markedly reduced the current density of BK when the stimulation voltage reached 50–150 mv (Figure 4E). Therefore, the E884K variant is also considered as a LOF variant.

### BK Channel Knockout Mediates Epilepsy

**FIGURE 5:**
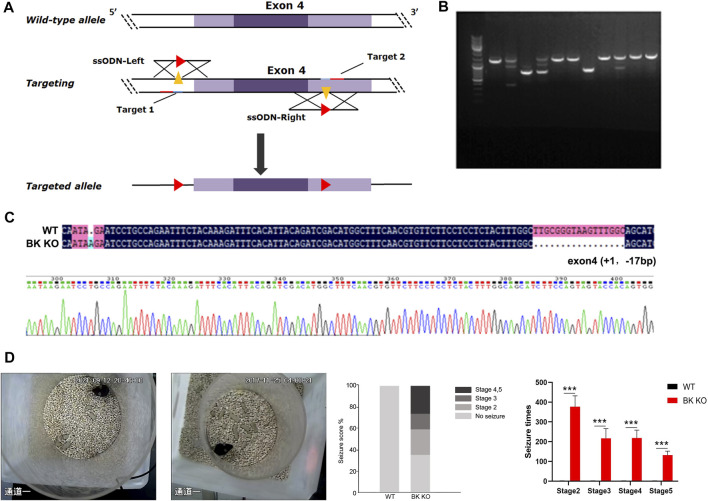
Construction of BK KO mice with spontaneous epilepsy. **(A)** Schematic outlining the generation of BK knockout mice using the CRISPR/Cas9 system. The targeting sites of KCNMA1 (gene encoding the α subunit of BK, BKα) are shown. **(B,C)** BK KO mice were established by breeding BK^+/−^ males and females. The targeted fragment of KCNMA1 was amplified by PCR using genomic DNA templates, and the BK channel deletion was confirmed by sequencing. Genome sequencing of BK KO mice showed a frameshift mutation (- 16 bp) in exon 4. **(D)** Spontaneous epileptic behavior of BK KO mice. The proportion of different seizure stages in BK KO/WT mice was observed for 2 h (***p < 0.001, *n* = 3).

BK channel knockout (BK KO, *kcnma1* ^−/−^) mice were generated by the deletion of exon four of *kcnma1* (gene encoding the α subunit of BK, BKα) using the CRISPR/Cas9 strategy (Figure 5A) (Wang et al., 2019). The BK KO mice, which carried a 16 bp fragment deletion in exon 4, was identified by PCR (Figure 5B) and confirmed by sequencing (Figure 5C). By observing movie (Supplementary Movies S1, S2), it is more intuitive to show that BK KO mice have an epileptic phenotype. Through continuous recording for 2 h, it was found that BK KO mice showed nearly 25% of grade 4–5 convulsive seizures (Figure 5D, *n* = 3). In addition, the seizure time and grade of BK KO mice were significantly greater than those of WT mice (Figure 5D, *p* < 0.001, *n* = 3). Thus, we found that BK KO mice have spontaneous epileptic symptoms, mainly manifested as generalized tonic clonic seizures and absence seizures (Figure 5D), which corresponds to the epileptic phenotype of human BK channel functional inactivation gene mutation (BK channel frameshift mutation) (Tabarki et al., 2016).

### FP Characteristics of BK KO Mice

**FIGURE 6:**
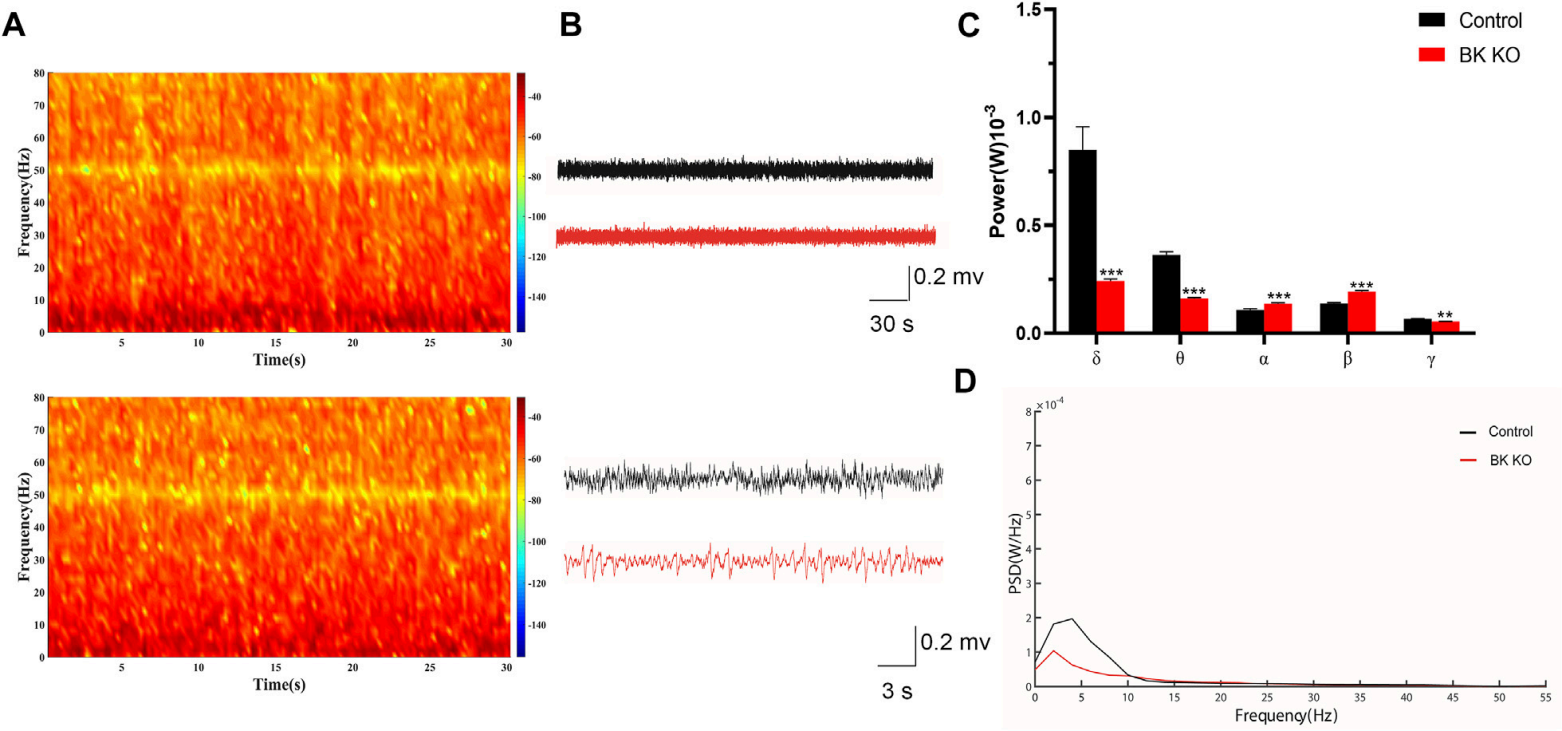
*In vivo* multichannel EEG recording of mice. **(A,B)** FP signals and spectral heat maps from a representative WT (black) and BK KO (red) mice are shown, respectively. **(C)** Spectral analysis of PSD values on different frequency δ, θ, α, β, and γ waves in each group. **(D)** The PSD of mice in each group (Compared with control group, *p < 0.05, **p < 0.01, ***p < 0.001, *n* = 4).

Then, to determine differences of BK KO and control mice in EEG levels, the power spectral density (PSD) of BK KO and control mice on was directly measured. We compared the field potential (FP) signals of BK KO and control mice, and differences in FP activity were visualized via using heat maps of spectral density generated by OmniPlex software (Plexon, United States). Compared with the control mice, BK KO mice had no significant differences on the FP activity (Figure 6A). The results showed that the EEG of WT mice presented basic waves with low frequency and amplitude, and there were no abnormal epileptic waves. The EEG of BK KO mice showed the basic wave with lower frequency and amplitude, accompanied by a small number of spike-waves (Figure 6B). The energy intensity values of five common rhythms collected by EEG were counted, δ Wave (0.5–4 Hz) belongs to a slow wave, which is the main rhythm in the sleep state of mice. Compared with control group, the δ rhythmic energy intensity of BK KO mice decreased significantly (Figure 6C, *p* < 0.001, *n* = 4). θ wave (4–7 Hz) and δ wave is similar to the rhythm that appears during sleep. θ rhythmic energy intensity in BK KO mice was lower than that in control group (Figure 6C, *p* < 0.001, *n* = 4). α wave (8–13 Hz) is the normal brain wave of mice. Compared with control group, α rhythmic energy intensity of BK KO mice increased significantly (Figure 6C, *p* < 0.001, *n* = 4). β wave (15–30 Hz) is the main rhythm when the brain is excited. Compared with control group, β rhythmic energy intensity in BK KO mice increased significantly (Figure 6C, *p* < 0.001, *n* = 4). γ wave (>30 Hz) belongs to a fast wave that occurs during rapid eye movement sleep and γ rhythmic energy intensity in BK KO mice decreased significantly (Figure 6C, *p* < 0.01, *n* = 4). Moreover, the PSD of total frequency waves of BK KO mice was obviously smaller than that of the control group (Figure 6D). Thus, the slow-wave power was reduced, normal and excited wave power was enhanced in BK KO mice.

### Motor Impairment in BK KO Mice

**FIGURE 7:**
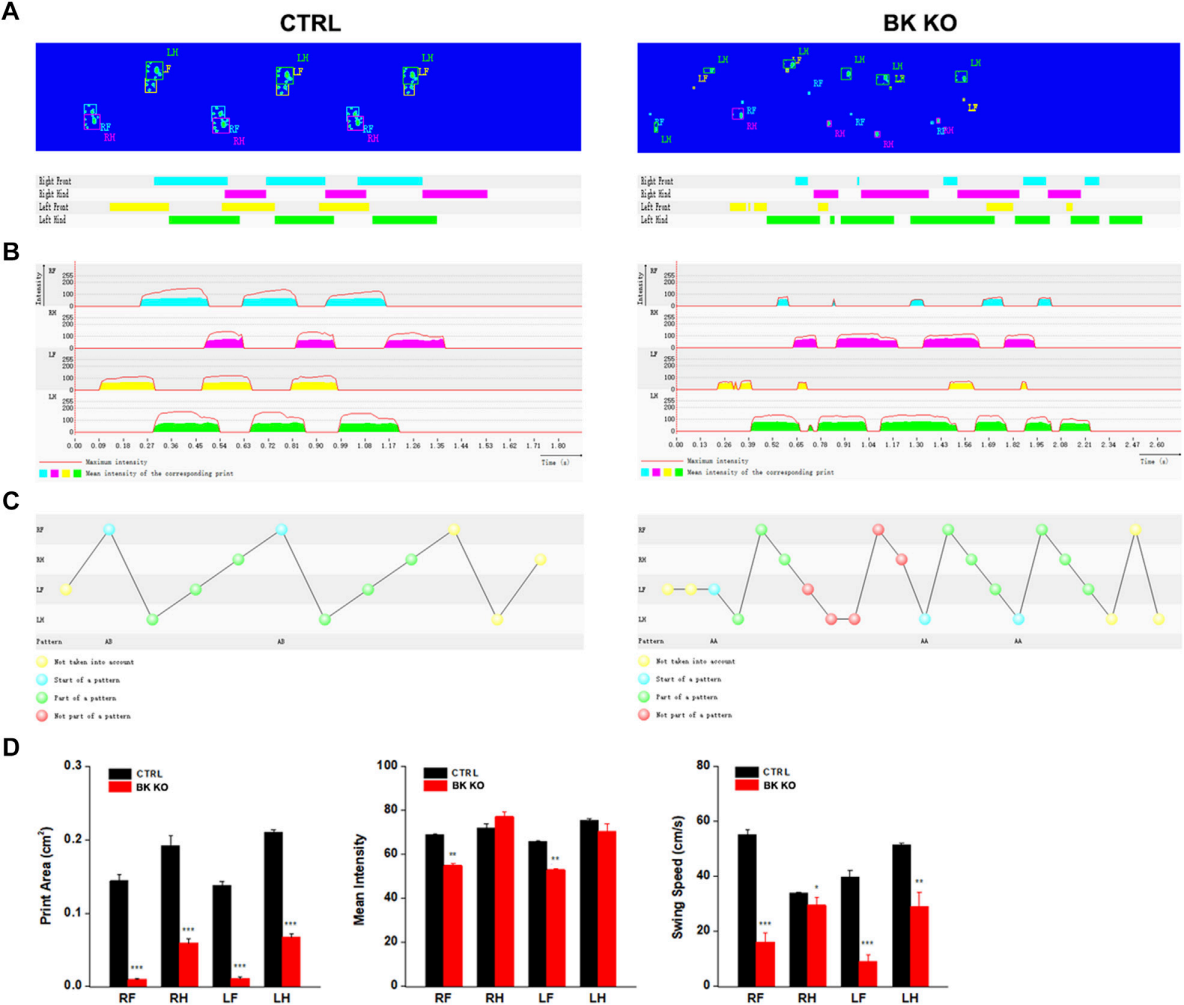
Gait analysis was performed by DigiGait imaging system. **(A–C)** Schematic diagram of WT and BK KO mouse footprints. **(D)** Print area (cm^2^), mean intensity and Swing speed (cm/s) of the right front (RF), the right hind (RH), the left front (LF), the left hind (LH) limb were chosen as the observation index. The Data are presented as means ± SEM. (Compared with control group, *p < 0.05, **p < 0.01, ***p < 0.001, *n* = 5).

Using the catwalk gait analysis system, a number of gait abnormalities were identified. By observing movie (Supplementary Movies S3, S4), it can be intuitively found that BK KO mice have abnormal gait and shorter stride phenotype (Figures 7A–C). The print area (RF, RH, LF, LH) of BK KO mice was narrower than that of control mice (Figure 7D, *p* < 0.001, *n* = 5). Moreover, the mean intensity (RF, LF) of BK KO mice less than that of control mice (Figure 7D, *p* < 0.01, *n* = 5). Swing speed and stride length were smaller, with an unsteady gait pattern in the BK KO mice, compared to the gait of control mice (Figure 7D, *n* = 5). Results revealed BK KO mice presented motor impairment.

In addition, the total distance (Supplementary Figure S2A, *p* < 0.001, *n* = 8), average speed (Supplementary Figure S2C, *p* < 0.001, *n* = 8) of BK KO mice significantly reduced in the open field, and the total distance (Supplementary Figure S2G, *p* < 0.001, *n* = 8) of BK KO mice markedly decreased in the *Y*-maze test. Thus, BK KO mice also showed motor impairment in the Y-maze and open field test. What’ more, BK KO mice showed smaller time in the central area (Supplementary Figure S2B, *p* < 0.001, *n* = 8), frequency and aim-quadrant stay time (Supplementary Figures S2D,E, *p* < 0.001, *p* < 0.05, *n* = 6), and the percentage of spontaneous alternation (Supplementary Figure S2F, *p* < 0.001, *n* = 8) as compared with the WT mice. Although motor impairment suggested that it might have an impact on anxiety and cognitive impairment of BK KO mice, there were also literatures supporting that BK KO have the cognitive impairment phenotype without the interference factor of locomotion (Sausbier et al., 2004). This strongly suggested that BK KO mice might be accompanied by anxiety and cognitive impairment in addition to the phenotype of motor impairment.

### Transcriptome Sequencing and Analysis of Hippocampus and Cortex of BK KO Mice

**FIGURE 8:**
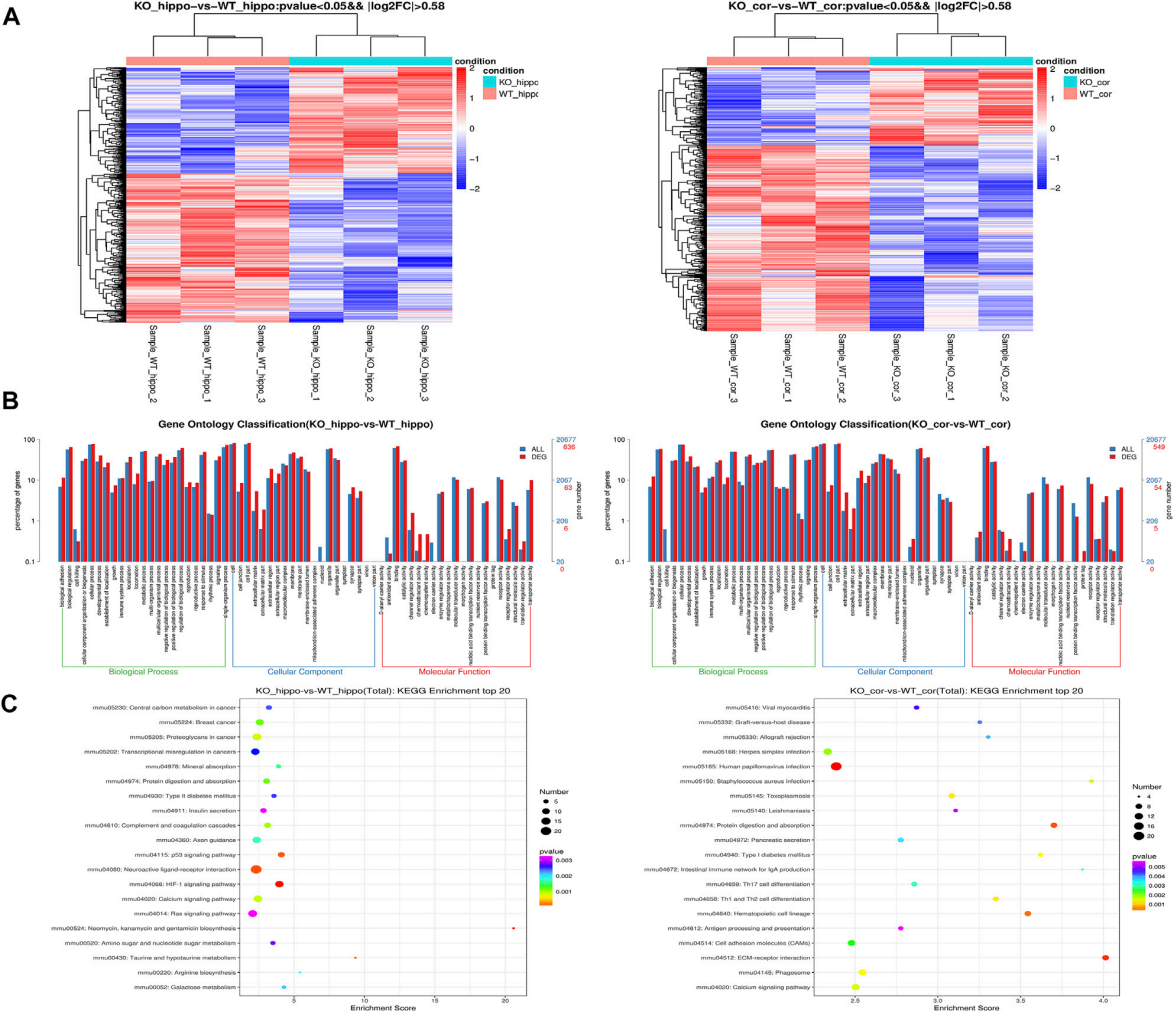
Transcriptome profiling in the hippocampus and cortex tissues of mice. **(A)** Heatmap of the DEGs. **(B)** Enriched Biological Process pathway, cellular component and molecular function in GO analysis (*p* < 0.05). **(C)** KEGG enrichment analysis of DEGs. The intensity of the color depends on the *p* value. The size of plot depends on the gene count. (*n* = 3).

In this study, we used RNA-Seq to analyze the transcriptome of BK KO/WT mice hippocampus and cortex, and performed transcriptome profiling to characterize the differentially expressed genes. A total of 652 genes were screened with the threshold of significance at *p* < 0.05 and |log2foldchange| > 0.58, among which 382 genes were down-regulated and 270 genes were up-regulated in hippocampus tissues. In cortex tissues, we detected a total of 561 differentially expressed genes with the threshold of significance at *p* < 0.05 and |log2foldchange| > 0.58, including 162 up-regulated genes and 399 down-regulated genes (Figure 8A).

In order to better understand the potential functions of differentially expressed genes, Gene Ontology (GO) enrichment analysis was carried out to assess the involved pathways. Biological process pathway in GO analysis results showed that development process and biological adhesion were enriched (Figure 8B). The results of kyoto encyclopedia of genes and genomes (KEGG) showed that insulin secretion, axon guidance, p53 signaling pathway, HIF-1 signaling pathway and calcium signaling pathway were enriched in hippocampus (Figure 8C). On the other hand, cortical KEGG results showed that cell adhesion molecules (CAMs), ECM-receptor interaction, phagosome and calcium signaling pathway were enriched (Figure 8C).

### Genes Were Verified

**FIGURE 9:**
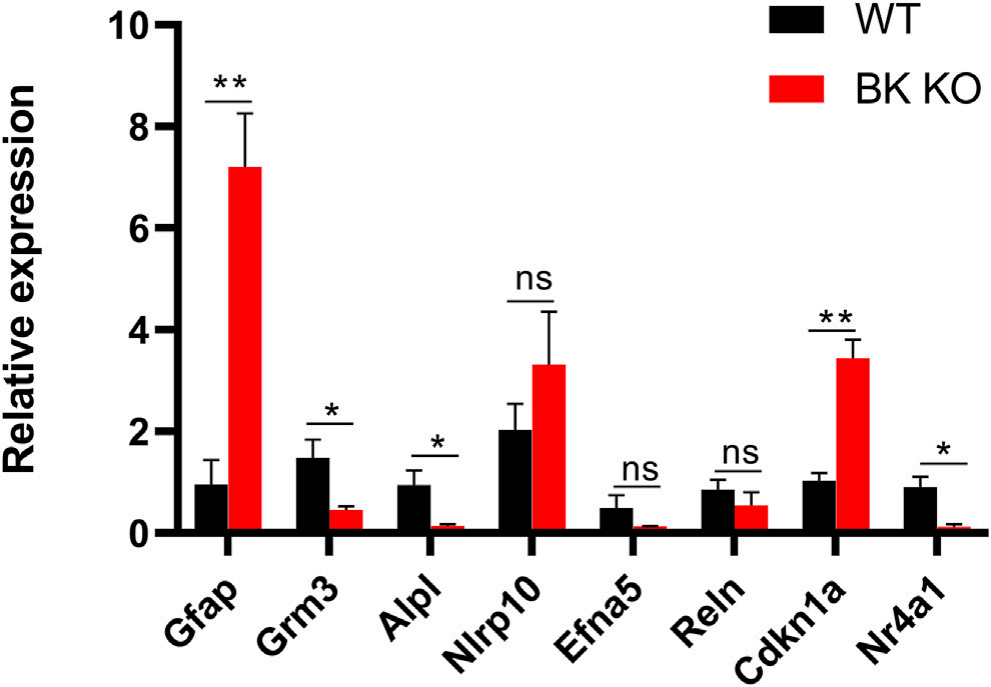
Differential expression of mRNAs between the cortex and hippocampus of WT (Black) and BK KO (Red) mice validated by RT-PCR. Gfap and Grm3 associated with astrocyte activation, Alpl, and Nlrp10 associated with neuroinflammation, Efna5 and Reln associated with epilepsy, Cdkn1a and Nr4a1 associated with autophagy. Gfap, Grm3, Nlrp10, Alpl in cortex, and Efna5, Reln, Cdkn1a, Nr4a1 in hippocampus. Ns (no significant difference, *p* > 0.05), ∗*p* < 0.05, ∗∗*p* < 0.01.

The transcriptomic results were compared with NCBI genebank (https://www.ncbi.nlm.nih.gov/gene/?term=), and it was found that DEGs in Supplementary Tables S1, S2 were closely related to epilepsy, astrocyte activation, neuroinflammation and microglia autophagy. Through RT-PCR, we used three groups of the cortex and hippocampus between WT and BK KO mice to verify the bold eight genes in the Supplementary Tables S1, S2. (Foldchange was larger in the same group of genes). Gfap and Cdkn1a gene highly expressed in BK KO mice (Figure 9, *p* < 0.01, *n* = 3). There were three genes that were lowly expressed in BK KO mice, Grm3, Alpl and Nr4a1 (Figure 9, *p* < 0.05, *n* = 3). In all, the results of RT-PCR and transcriptomics were highly consistent. We continued to explore the possible mechanisms of epilepsy through transcriptome.

### Abnormal Autophagy in BK KO Mice

Autophagy is a normal catabolic process in cells. Various types of biological macromolecules undergo degradation and circulation through lysosomal digestion to maintain cell homeostasis. Improving the neuroinflammation response in the pathogenesis of multiple sclerosis (MS) can be achieved by enhancing autophagy (Liang and Le, 2015; Feng et al., 2017). This suggests that neuroinflammation and autophagy can influence each other, which in turn affects the progression of CNS-related diseases.

**FIGURE 10:**
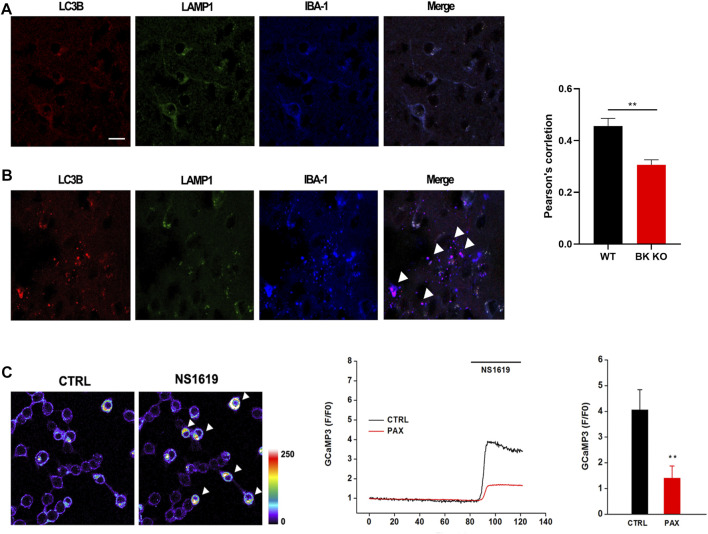
Autophagy in BK KO mice was abnormal. Calcium imaging found that the activation of BK channel could activate lysosomal trpml1 (autophagy key calcium channel). **(A)** Co labeling of microglia, LC3B, IBA-1, and LAMP1 in control mice. **(B)** Co labeling of microglia, LC3B, IBA-1, and LAMP1 in BK KO mice. **(C)** NS1619 was applied to HEK293T transfected with BK channel and TRPML1-GCaMP3 in order to detect its regulation of lysosomal calcium outflow (^∗∗^ *p* < 0.01, *n* = 6).

Transcriptomics results showed that there were 11 differentially expressed genes (DEGs) related to microglia autophagy in hippocampus and 5 DEGs related to microglia autophagy in cortical tissues (Supplementary Table S2). In order to further explore the role of autophagy in epilepsy, immunofluorescence experiments were performed on the hippocampus of WT and BK KO mice. In the hippocampal CA3 region of Ctrl group mice, autophagosome marker LC3B, lysosome marker LAMP1 and microglia marker IBA-1 were co-labeled, indicating that the interaction and fusion of autophagosome and lysosome in hippocampal microglia was normal (Figure 10A), but in the hippocampus CA3 region of BK KO mice, LC3B, LAMP1 and IBA-1 were partially not co-labeled, indicating abnormal interaction and fusion of autophagosomes and lysosomes in microglia of BK KO mice (Figure 10B). During epilepsy, there may be abnormal interaction and fusion between autophagosomes and lysosomes in hippocampal microglia. TRPML1 (key calcium channel of autophagy) promotes the fusion of autophagosomes and lysosomes, and the lysosomal calcium release of TRPML1 is closely related to autophagy (Scotto Rosato et al., 2019; Zhang et al., 2019). BK channel and TRPML1-GCaMP3 were co-expressed in HEK293T. The opener NS1619 of BK channel could induce the calcium outflow of lysosomes. Paxilline (PAX), a specific inhibitor of BK channel, can significantly inhibit the lysosomal calcium outflow (Figure 10C). In general, the activation of BK channel could activate lysosomal TRPML1. It suggested that BK might regulate the autophagy pathway from TRPML1.

## Discussion

In this study, we identified three KCNMA1-LOF mutants (E155Q, R458T, E884K), of which E155Q was a *de novo* mutant. All three variants showed profound effects on BK channel function, and played its role through LOF mechanism (BK channel current decreased and I-V curve shifted to the positive voltage direction). Faster BK current activation directly increases neuronal firing rate by causing faster repolarization of action potentials (Jaffe et al., 2011; Contet et al., 2016), which might be the cause of BK-GOF mediated epilepsy. The causes of BK-LOF mediated epilepsy include the inhibition of repolarization of action potential, resulting the increase of neuronal excitability, and the role of neuroimmune inflammation. What’s more, hβ4 had no effect on the I-V curve and current amplitude density of E155Q mutant, but activation time constant (*τ*) of E155Q+β4 channel was greater than that of E155Q mutant (Supplementary Figure S1).

Compared with wild-type littermates, *kcnma1* ^−/−^ mice lost weight, interestingly, so did the *kcnma1* ^+/−^ mice. Susan T Halm et al. speculated that the lack of *kcnma1* allele leads to insufficient grip in mice, which may limited the cubs’ access to nutrition (Halm et al., 2017). It was worth noting that the fertility of adult BK KO mice decreased and failed without exception in the process of 15 mating (Transcriptomics results suggested that Adam18, Cabyr and other genes related to sperm function were abnormal, and Eqtn regulated the abnormality of sperm and egg plasma membrane fusion). In addition, the results of KEGG (Figure 8C) showed that insulin secretion was enriched, which provided possible evidence for the imbalance of body weight and fat in BK KO mice (Halm et al., 2017). Disturbance of insulin release may damage health and cause signals to convert energy for growth into fat storage. Of note, the malnutrition and developmental delay caused by the weak grip of BK KO mice are reminiscent of the developmental delay found in the patient with the KCNMA1-LOF (E155Q) variant.

Although the epileptic phenotypes of BK KO mice were similar with those of clinical patients, such as developmental delay and interictal epileptiform discharge (IED), the FP characteristics of BK KO mice were different from those of clinical patients. Specifically, the intensity of δ and θ energy rhythms in BK KO mice reduced, while rhythmic energy intensity of δ and θ increased in the patient carrying the E155Q mutation site. We guess that it is mainly caused by the difference in detection method and detection area. In clinical testing, non-invasive EEG recording is mainly used to detect the membrane potential of neurons in the cortex, and we use *in vivo* multichannel electrophysiological recording to detect the local field potential of the hippocampus in animals. In addition, abnormal background activity amplitude may also affect IED. Christine M. Muheim et al. pointed out that the delta slow wave power of BK KO mice was reduced in cortex (<4 Hz) (Muheim et al., 2019). In our experiment, in addition to the decrease of delta slow wave power, the power of θ wave (4–7 Hz) and γ wave (>30 Hz) also decreased. Of note, the power of α wave and β wave increased, which may be the cause of spontaneous epilepsy in BK KO mice. In the motor cortex, beta waves are mainly involved in grasping, muscle contraction and maintaining attention (Khanna and Carmena, 2015). The abnormal up regulation of β wave may lead to abnormal excitation of neurons, excessive increase of motor control ability, and then lead to motor dysfunction.

In addition, we found that BK KO mice have a dyskinetic phenotype through a series of behavioral experiments. And BK KO mice showed smaller time in the central area (Supplementary Figure S2B, *p* < 0.001, *n* = 8), frequency and aim-quadrant stay time (Supplementary Figures S2D,E, *p* < 0.001, *p* < 0.05, *n* = 6), and the percentage of spontaneous alternation (Supplementary Figure S2F, *p* < 0.001, *n* = 8). The frequency of seizures and the duration of the disease have a negative impact on cognitive impairment (Jokeit and Ebner, 1999; Allone et al., 2017). Although, motor impairment might have an impact on anxiety and cognitive impairment of BK KO mice, one study cleverly excluded interference of motor, proving that BK KO mice have the cognitive impairment phenotype (Sausbier et al., 2004). This strongly suggested that lacking *kcnma1* genes (BK KO) may cause anxiety and cognitive impairment in BK KO mice, which required further study.

We further explored the possible molecular mechanisms of BK-LOF-mediated epilepsy through transcriptomics in the hippocampus and cortex. Gene Ontology (GO) analysis showed that DEGs were related to protein labeling, protein binding transcription factor activity, development process and biological adhesion (Figure 8B). The kyoto encyclopedia of genes and genomes (KEGG) showed these DEGs were mainly enriched in insulin secretion, axon guidance, p53 signaling pathway, HIF-1 signaling pathway, calcium signaling pathway, cell adhesion molecules (CAMs), ECM-receptor interaction, and phagosome (Figure 8C). In the process of epilepsy, including the changes of gene expression, neuroinflammation, protein production and connection, these may be the targets of inhibiting epilepsy. The results of KEGG showed that Cdkn1a is closely related to the HIF-1 signaling pathway, and dysregulated HIF-1 signaling may play a role in the pathogenesis of epilepsy in hippocampus (Merelli et al., 2018). A large number of glial cells activate and proliferate, glutamate and the secretion of inflammatory factors increases, which reduces the convulsion threshold and increases the excitability of brain neurons, and accelerates spontaneous recurrent convulsions (Friedman and Dingledine, 2011; Huberfeld et al., 2015). Epilepsy like activity *in vitro* and prolonged seizures *in vivo* lead to increased p53 accumulation and transcriptional activity (Sakhi et al., 1994; Liu et al., 1999; Tan et al., 2002; Araki et al., 2004). Abnormal axon guidance may induce mossy fiber germination, and the “wrong” guidance of mossy fiber may be a necessary process of dentate nerve circuit homeostasis under the condition of epilepsy (Koyama and Ikegaya, 2018). Meanwhile, the mammalian target of rapamycin (mTOR) is inhibited by rapamycin to prevent mossy fiber sprouting and reduce seizures in rodent models of acquired epilepsy (Zeng et al., 2009). Cell adhesion molecules (CAMs) may form trans synaptic complexes that are essential to correctly identify synaptic partners and further for determine the establishment and dynamics of synapses (Gorlewicz and Kaczmarek, 2018). Dysfunction of transsynaptic adhesion is associated with epilepsy (Gorlewicz and Kaczmarek, 2018). This strongly suggested that the DEGs find in transcriptomics, particularly those related to astrocyte activation, neuroinflammation and autophagy, may be the molecular mechanism of BK-LOF mediated epilepsy.

In summary, we identified and functionally characterized three different LOF variants in the BK channel (E155Q, R458T, E884K), of which E155Q variant was a *de novo* mutant and affected one patient. All the above variants caused a positive shift of the I-V curve and played a role through the loss-of-function (LOF) mechanism. Moreover, the β4 subunit slowed down the activation of the E155Q mutant. BK KO mice had spontaneous epilepsy, motor impairment, autophagic dysfunction, abnormal electroencephalogram (EEG) signals, as well as possible anxiety and cognitive impairment. In addition, BK might regulate the autophagy pathway from TRPML1. Dysregulation of gene expression, especially astrocyte activation, neuroinflammation and autophagy, might be the molecular mechanism of BK-LOF meditated epilepsy.

## Data Availability

The data presented in the study are deposited in the GEO repository, accession number GSE191038.
