# Supplementary material for: Molecular Mechanisms of Epileptic Encephalopathy Caused by KCNMA1 Loss-of-Function Mutations
Source: Front Pharmacol. 2022 Jan 13;12:775328. doi: 10.3389/fphar.2021.775328 (PMC8793784; doi:10.3389/fphar.2021.775328)
Supplement: Supplementary file 3 [file DataSheet1.PDF]

Supplementary Material

Molecular Mechanisms of Epileptic Encephalopathy Caused by KCNMA1 Loss-of-function Mutations

Yao et al

supplementary movie S1 WT.avi

supplementary movie S2 BK KO.a

supplementary movie S3 WT.mp4

supplementary movie S4 BK KO.r

Supplementary Movie 1-4. (1-2) The representative behavior of WT and BK KO mice is in the epilepsy behavior observation. (3-4) Representative gait of WT and BK KO mice.

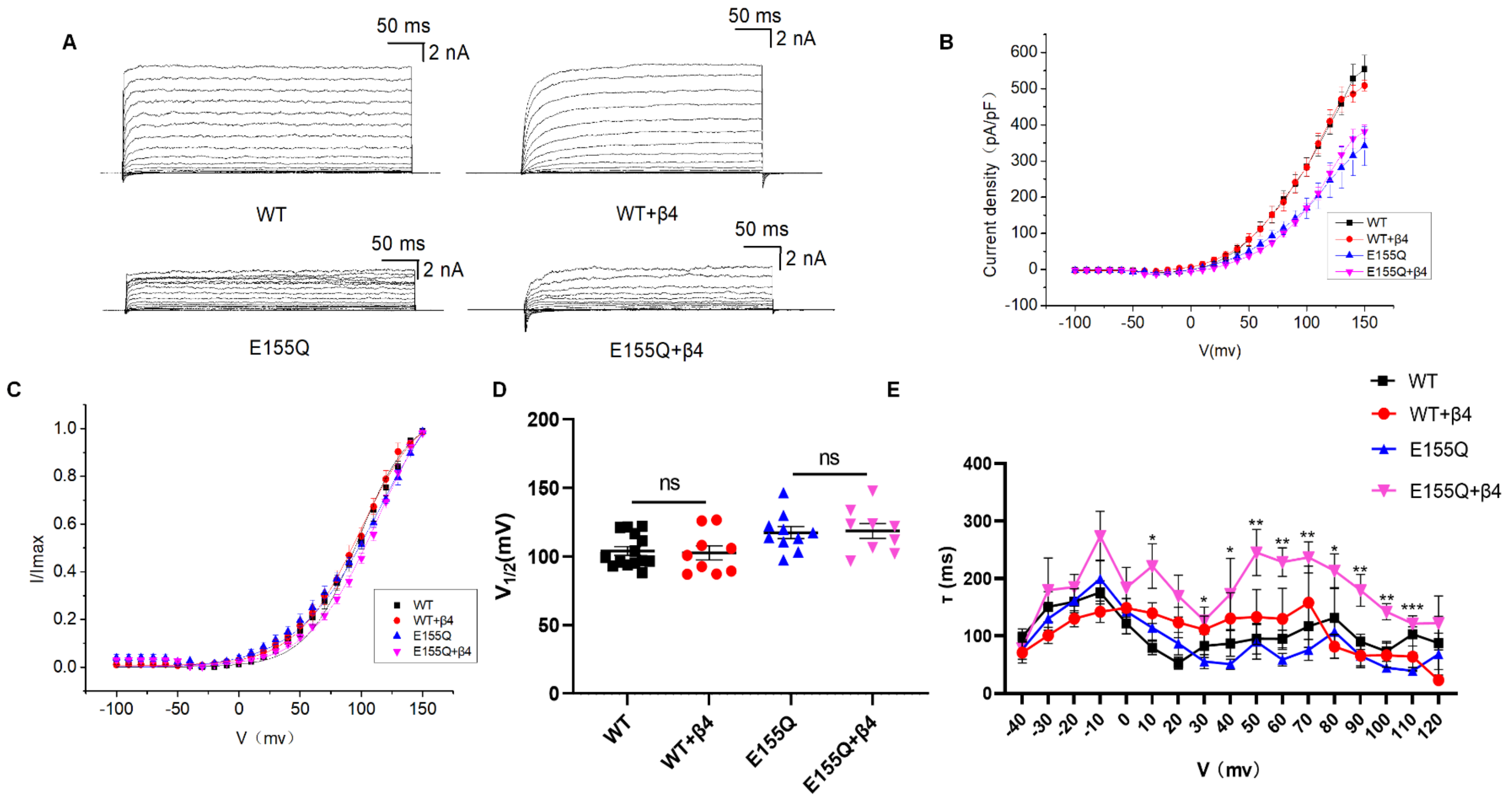

Supplementary Figure 1. Electrophysiological characterization of WT, E155Q, WT+β4, and E155Q+β4. (A) Representative macroscopic currents of WT, E155Q, WT+β4, and E155Q+β4 from whole-cell patch experiments in the presence of 1 μm Ca^{2+}. (B) The current density of WT, E155Q, WT+β4, and E155Q+β4 are shown at 1 μm Ca^{2+}. The data are presented as mean ± SEM. (C) The G-V curves of WT, E155Q, WT+β4, and E155Q+β4 are shown at 1 μm Ca^{2+}. The G-V curves are fitted by Boltzmann function (solid lines) with V1/2 and slope factor at nominal at 1 μm Ca^{2+} [97.5 ± 4.1 mV, 21.4 ± 2.1 WT, 98.3 ± 1.7 mV, 26.0 ± 0.9 WT+β4, 113.4 ± 6.4 mV, 33.2 ± 3.3 p.(E155Q), 113.3 ± 5.7 mV, 27.4 ± 2.3 E155Q+β4]. (D) Scatter plots of voltage at half-maximal activation (V1/2) for WT and variants. (E) Activation rate (τ) of macroscopic WT, WT+β4, E155Q, E155Q+β4 channel currents were obtained by fitting the individual current traces to simplex single exponential function in the presence of intracellular 1 μm free calcium. (Compared with E155Q, *$P < 0.05$, **$P < 0.01$, ***$P < 0.001$, n= 6-9/group). Ns (no significant difference, $P > 0.05$)

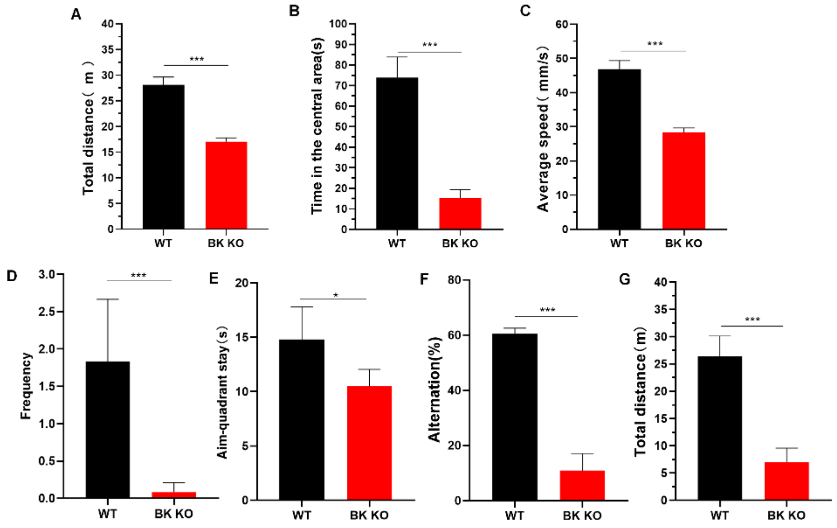

Supplementary Figure 2. A series of behavioral experiments. (A-C) The results of WT and BK KO mice in open field. (n=8) (D-E) The results of WT and BK KO mice in morris water maze. (n=8) (F-G) The results of WT and BK KO mice in Y-maze. (Compared with WT mice, *$P < 0.05$, **$P < 0.01$, ***$P < 0.001$, n=6)

Supplementary Table 1. DEGs closely related to epilepsy, astrocyte activation and neuroinflammation.

	gene_id	baseMean	baseMean_control_WT_	baseMean_case_KO_	foldChange	log2FoldChange	pval	up_do
Epilepsy	**Reln**	**2457.355**	**1535.016**	**3379.693**	**2.201731**	**1.138638**	**1.82E-05**	**Up**
	Grin2b	822.1808	630.4744	1013.887	1.608134	0.685387	0.040213	Up
	**Efna5**	**563.0771**	**696.9329**	**429.2214**	**0.615872**	**-0.6993**	**0.029644**	**Down**
Astrocyte activation	**Gfap**	**6102.595**	**4068.559**	**8136.631**	**1.99988**	**0.999914**	**0.010179**	**Up**
	Mt2	3103.599	2335.686	3871.512	1.657548	0.729051	0.015744	Up
	**Grm3**	**1047.238**	**1285.782**	**808.6943**	**0.628952**	**-0.66898**	**0.000179**	**Down**
	Ednrb	960.446	1153.981	766.9104	0.664578	-0.58949	0.004801	Down
	Gjb1	177.023	213.6292	140.4169	0.657293	-0.60539	0.011158	Down
	Itgb4	238.7636	293.8384	183.6889	0.625136	-0.67776	0.005983	Down

Neuroinflammation	**Nlrp10**	**10.22872**	**3.301993**	**17.15545**	**5.195484**	**2.377258**	**0.020209**	**Up**
	**Alpl**	**188.9666**	**246.8251**	**131.1082**	**0.531179**	**-0.91273**	**9.66E-05**	**Down**
	Il33	759.7587	964.7661	554.7512	0.575011	-0.79834	2.38E-06	Down
	Klf4	186.6469	235.4127	137.8811	0.5857	-0.77177	0.006209	Down
	Neil1	182.5501	233.6232	131.4771	0.562774	-0.82937	0.005744	Down

Supplementary Table 2. DEGs associated with microglia autophagy.

	gene_id	baseMean	baseMean_control_WT_	baseMean_case_KO_	foldChange	log2FoldChange	pval	up_down
Microglia autophagy in hippocampus	**Cdkn1a**	**968.518**	**605.9227**	**1331.113**	**2.196837**	**1.135428**	**0.001467**	**Up**
	Cxcr4	37.81871	23.1991	52.43833	2.260361	1.176553	0.020476	Up
	Brca1	44.23256	29.16251	59.30261	2.033522	1.023981	0.020235	Up
	Spp1	315.7994	221.4545	410.1444	1.852048	0.889121	0.010182	Up
	Mt1	6333.231	4698.986	7967.475	1.695573	0.761773	0.000526	Up
	Il6ra	147.8406	115.8498	179.8313	1.55228	0.634388	0.032046	Up
	Ucp2	637.7879	500.8519	774.7239	1.546812	0.629298	0.014745	Up
	Bid	178.7005	142.4302	214.9707	1.509305	0.593885	0.027031	Up
	P2rx4	261.1353	313.6619	208.6087	0.665075	-0.58841	0.012902	Down

	**Nr4a1**	**1849.143**	**2557.169**	**1141.116**	**0.446242**	**-1.1641**	**0.015791**	**Down**
	Cryab	1334.602	1672.73	996.4745	0.595718	-0.7473	0.017248	Down
Microglia autophagy in cortex	Cdkn1a	1186.845	796.3142	1577.375	1.980845	0.986116	1.47E-05	Up
	Il33	759.7587	964.7661	554.7512	0.575011	-0.79834	2.38E-06	Down
	Klf4	186.6469	235.4127	137.8811	0.5857	-0.77177	0.006209	Down
	Mt1	6615.389	4846.004	8384.775	1.730245	0.790977	0.01027	Up
	Ucp2	620.1966	493.9267	746.4665	1.51129	0.595781	0.015063	Up

Supplementary Table 3. List of the PCR primer sequences.

Gene Symbol	Refseq Accession	Forward Primer (5'-3')	Reverse Primer (5'-3')	Product Size (bp)
Gfap	NM_010277.3	GAGGGACAACTTTGCACAGG	TCCTCCAGCGATTCAACCTT	165 bp
Grm3	NM_181850	CTGGAGGCCATGTTGTTTGC	CATCCACTTTAGTCAACGATGCT	160 bp
Alpl	NM_001287172.1	CGCCATGACATCCCAGAAAG	ACCGAATGTGAAAACGTGGG	70 bp
Nrlp10	NM_175532	GGCTGAAGAAGAAGATGGCG	GCTGTCTCCATTGCCATCAG	116 bp
Efna5	NM_010109	TTCTGGTGCTCTGGATGTGT	AGCGCTCAGTCTTGTCTTCT	198 bp
Reln	NM_001310464	ATGTCATTCCTGGAGCCACA	GCTTGCAAGTCTATGGAGCC	200 bp
Cdkn1a	NM_001111099.2	ACAAGAGGCCCAGTACTTCC	GTTTTCGGCCCTGAGATGTT	141bp
Nr4a1	NM_010444.2	CTTGAGTTCGGCAAGCCTAC	GGTGTCAAACTCTCCGGTGT	117bp

Supplementary Table 4. The KEGG of HIF-1 signaling pathway, calcium signaling pathway, neuroactive ligand-receptor interaction, insulin secretion, axon guidance, mTOR signaling pathway, axon guidance, cell adhesion molecules, and ECM-receptor interaction, phagosome.

KEGG	gene	baseMean	baseMean_control_ WT	baseMean_case_ KO	foldChange	log2FoldChange
HIF-1 signaling pathway (in hippocampus)	Hk3	32.57380806	45.45081714	19.69679897	0.433365123	-1.206345043
	Hkdc1	245.0439431	337.0487673	153.039119	0.454056308	-1.139056875
	Tek	274.5099973	353.0070247	196.0129699	0.555266485	-0.848747774
	Rps6kb2	535.8660862	658.1870498	413.5451226	0.628309419	-0.670452888
	Akt2	1854.31584	2260.714242	1447.917437	0.640469021	-0.642799305
	Arnt	739.4528171	900.7711032	578.134531	0.6418218	-0.639755302
	Angpt2	69.19384456	32.34674079	106.0409483	3.278257585	1.712929217
	Serpine1	38.18349293	21.34536351	55.02162235	2.577684954	1.366075948
	Cdkn1a	968.5180257	605.9226955	1331.113356	2.196836933	1.135427785
	Hk2	126.7332794	91.77701064	161.6895481	1.76176525	0.817021703
	Camk2d	2644.613486	1981.045977	3308.180996	1.669916314	0.739775805
	Il6ra	147.8405644	115.8498178	179.831311	1.552279619	0.63438846

Calcium signaling pathway (in hippocampus)	Adcy4	78.25392412	97.66963506	58.83821318	0.602420733	-0.731156671
	Atp2a3	213.8816199	260.6393582	167.1238817	0.641207386	-0.64113705
	Cacna1s	20.18585108	9.619380418	30.75232174	3.196912941	1.676679457
	Tacr1	174.78959	90.85851136	258.7206687	2.84751164	1.50970174
	Plce1	322.1630866	191.4356173	452.890556	2.365759112	1.242303182
	F2r	157.536312	101.0267972	214.0458269	2.11870348	1.083181692
	Pde1c	285.4972114	197.1446999	373.8497229	1.896321449	0.923203539
	Oxtr	285.4972114	197.1446999	373.8497229	1.896321449	0.923203539
	Tacr3	59.16805166	43.16634515	75.16975816	1.741397329	0.800245416
	Nos1	1844.555695	1366.632278	2322.479112	1.699417722	0.765040515
	Camk2d	2644.613486	1981.045977	3308.180996	1.669916314	0.739775805
	Htr2a	281.61688	219.3711514	343.8626085	1.567492381	0.648458431
Neuroactive ligand-receptor interaction (in hippocampus)	Gm10334	6.502863169	11.77340594	1.232320398	0.10466983	-3.256082434
	Rxfp1	367.3026768	558.5753768	176.0299768	0.315140953	-1.665930847
	Apln	544.5619505	772.6738175	316.4500835	0.409551969	-1.287881564

	Grm3	884.7714502	1090.191555	679.351345	0.623148603	-0.682351849
	S1pr1	2706.60169	3292.837767	2120.365613	0.643932609	-0.635018385
	P2rx4	261.1352599	313.6618604	208.6086594	0.665074992	-0.58841107
	Gh	155.9931244	0	311.9862488	Inf	Inf
	Gpr50	10.83626257	3.397636622	18.27488851	5.378706008	2.427259136
	Trh	140.0650476	54.96743336	225.1626618	4.096292078	2.034318587
	Tacr1	174.78959	90.85851136	258.7206687	2.84751164	1.50970174
	Gabrq	55.73090014	29.4278087	82.03399158	2.78763507	1.47904171
	Tac1	1050.395889	597.299164	1503.492614	2.517151713	1.331792173
	C3ar1	55.0193789	33.90031179	76.138446	2.245951202	1.167326582
	F2r	157.536312	101.0267972	214.0458269	2.11870348	1.083181692
	P2ry1	110.9114544	78.95154442	142.8713645	1.809608229	0.855677395
	Oxtr	221.2229563	161.1545644	281.2913483	1.745475527	0.80362013
	Tacr3	59.16805166	43.16634515	75.16975816	1.741397329	0.800245416
	Grin2b	822.1807872	630.4744162	1013.887158	1.608133704	0.68538736
	Gpr83	746.8594955	573.9019864	919.8170045	1.602742326	0.680542501
	Htr2a	281.61688	219.3711514	343.8626085	1.567492381	0.648458431
Insulin secretion (in hippocampus)	Kcnma1	3049.982763	4888.272414	1211.693111	0.247877575	-2.012300336

Pde4c	59.76669496	79.25323843	40.2801515	0.508246127	-0.976400778
Sirt4	367.1114034	476.7721663	257.4506404	0.539986724	-0.889004156
Abcc8	720.8844604	919.992383	521.7765379	0.567153106	-0.818189843
Stx1a	4540.736007	5713.914198	3367.557816	0.589360935	-0.762776658
Adcy4	78.25392412	97.66963506	58.83821318	0.602420733	-0.731156671
Trpm4	306.3017048	379.8715359	232.7318737	0.612659417	-0.706842806
Efna5	563.0771397	696.932901	429.2213784	0.615871884	-0.699297829
Casr	24.99927094	5.827595676	44.1709462	7.579617506	2.922125047
Trh	140.0650476	54.96743336	225.1626618	4.096292078	2.034318587
Cacna1s	20.18585108	9.619380418	30.75232174	3.196912941	1.676679457
Prox2	133.5822265	181.0039258	86.16052714	0.476014687	-1.070922007
Pde1c	285.4972114	197.1446999	373.8497229	1.896321449	0.923203539
Nnat	3312.094191	2301.238784	4322.949599	1.878531523	0.909605326
Nos1	1844.555695	1366.632278	2322.479112	1.699417722	0.765040515
Camk2d	2644.613486	1981.045977	3308.180996	1.669916314	0.739775805
Nov	2257.273122	1713.924667	2800.621577	1.634040066	0.708443359
Tiam1	2870.310263	2187.263683	3553.356844	1.624567203	0.700055424

	Ucp2	637.7879378	500.8519451	774.7239305	1.546812263	0.629298107
axon guidance (in hippocampus)	Plxnb3	493.1069517	596.3267266	389.8871768	0.653814695	-0.613046292
	Robo3	633.0265091	904.7282148	361.3248034	0.399373864	-1.324188171
	Ephb2	353.696644	469.6821891	237.7110988	0.506110524	-0.982475621
	Etv4	196.9172029	254.7119086	139.1224972	0.546195496	-0.872510678
	Pak7	822.2467305	1056.339414	588.154047	0.556785101	-0.844807489
	Ssh3	835.9689124	1059.61791	612.3199149	0.577868597	-0.791186623
	Ephb3	532.5720576	668.542786	396.6013292	0.593232531	-0.753330381
	Efna5	563.0771397	696.932901	429.2213784	0.615871884	-0.699297829
	Sema7a	4446.368694	5494.386048	3398.351339	0.61851339	-0.693123267
	Fezf2	537.9627841	656.7196928	419.2058755	0.638333036	-0.64761878
	Ptpro	821.9230142	380.0070603	1263.838968	3.325830228	1.733714526
	Cxcl12	1319.000399	1756.791239	881.2095589	0.501601749	-0.995385715
	Cdh4	668.953444	396.4116768	941.4952112	2.375044092	1.247954297
	Cxcr4	37.81871071	23.19909571	52.4383257	2.260360763	1.176553051
	Reln	2457.354748	1535.016239	3379.693257	2.201731273	1.138638395
	Sirt4	367.1114034	476.7721663	257.4506404	0.539986724	-0.889004156

	Sema3d	2457.354748	1535.016239	3379.693257	2.201731273	1.138638395
	Camk2d	2644.613486	1981.045977	3308.180996	1.669916314	0.739775805
	Zswim5	389.094273	299.1579867	479.0305593	1.601262813	0.679210114
	Bcl11b	2746.909045	2115.163866	3378.654225	1.597348687	0.675679275
mTOR signaling pathway (in hippocampus)	Wnt9a	304.9762255	392.9107341	217.0417169	0.552394471	-0.856229216
	Rps6kb2	535.8660862	658.1870498	413.5451226	0.628309419	-0.670452888
	Akt2	1854.31584	2260.714242	1447.917437	0.640469021	-0.642799305
	Wnt10b	51.80985699	34.96167046	68.65804351	1.963809011	0.973654628
	Fzd5	78.85185412	54.32289688	103.3808114	1.903079867	0.928336109
	Rps6ka6	112.0705969	85.06150092	139.0796928	1.635048657	0.709333569
cell adhesion molecules (in cortex)	H2-Aa	70.33483203	98.26538095	42.40428311	0.431528201	-1.212473249
	H2-Eb1	77.52983085	106.8448628	48.21479889	0.45125987	-1.147969608
	H2-M10.2	8.634999259	14.34200807	2.92799045	0.204154846	-2.292264281
	H2-Q6	33.4020826	45.57710989	21.22705531	0.465739389	-1.102405194
	H2-T24	182.7787081	219.6481235	145.9092927	0.664286543	-0.590122406

	Itga4	265.9683448	323.6664733	208.2702163	0.643471702	-0.636051391
	Itgal	48.61361236	61.52735181	35.69987291	0.580227685	-0.785308961
	Itgam	553.4674993	669.6187426	437.3162561	0.653082461	-0.614662932
	Nectin3	382.3567359	461.4696201	303.2438517	0.65712636	-0.605757279
	Cldn3	12.26502008	5.372475681	19.15756448	3.565872722	1.834255209
ECM-receptor interaction (in cortex)	Thbs3	281.9628823	359.347752	204.5780125	0.569303721	-0.812729564
	Tnxb	124.6466925	155.6975401	93.59584486	0.60113888	-0.734229762
	Col6a4	65.79493816	108.8414435	22.7484328	0.209005247	-2.258388933
	Itga10	129.8554175	162.8464497	96.86438538	0.59482037	-0.749474042
	Itga4	265.9683448	323.6664733	208.2702163	0.643471702	-0.636051391
	Itgb4	238.7636445	293.8384165	183.6888725	0.62513566	-0.677758793
	Lama3	146.2019864	191.6536314	100.7503413	0.525689707	-0.927716608
	Gp1ba	27.02046155	17.19307495	36.84784815	2.143179638	1.09975278
phagosome (in cortex)	Synpo2	166.0171908	122.2646575	209.769724	1.715702053	0.778799038
calcium signaling pathway (in cortex)	Tnnc2	62.34934952	121.0672103	3.631488769	0.029995643	-5.059103251
	Adcy4	85.05976512	112.1306164	57.9889138	0.517155043	-0.951331229

Atp2a1	141.4462744	260.3761851	22.51636381	0.08647628	-3.531551731
Atp2a3	199.3719435	248.8383835	149.9055034	0.602421143	-0.73115569
Ednra	90.45202392	109.223362	71.68068581	0.656276134	-0.607625125
Ednrb	960.4459637	1153.981492	766.9104357	0.664577761	-0.589490079
Mylk2	5.242211118	10.48442224	0	0	#NAME?
Plcz1	14.17746477	22.78947936	5.565450181	0.244211379	-2.033797671
Ryr1	421.4672172	579.6279435	263.3064909	0.454268111	-1.138384062
Ryr3	669.4798872	811.6279474	527.331827	0.64972113	-0.62210747
Adra1b	721.5380363	491.9865234	951.0895491	1.933161792	0.950962386
